# The first draft genome of the aquatic model plant *Lemna minor* opens the route for future stress physiology research and biotechnological applications

**DOI:** 10.1186/s13068-015-0381-1

**Published:** 2015-11-25

**Authors:** Arne Van Hoeck, Nele Horemans, Pieter Monsieurs, Hieu Xuan Cao, Hildegarde Vandenhove, Ronny Blust

**Affiliations:** Biosphere Impact Studies, SCK•CEN, Boeretang 200, 2400 Mol, Belgium; Department of Biology, University of Antwerp, Groenenborgerlaan 171, 2020 Antwerp, Belgium; Centre for Environmental Research, University of Hasselt, Universiteitslaan 1, 3590 Diepenbeek, Belgium; Microbiology, SCK•CEN, Boeretang 200, 2400 Mol, Belgium; Leibniz Institute of Plant Genetics and Crop Plant Research (IPK), OT Gatersleben, Corrensstrasse 3, 06466 Stadt Seeland, Germany

**Keywords:** *Lemna minor*, Whole-genome sequencing, Duckweed, Biomass production, Ecotoxicology, Toxicogenomics

## Abstract

**Background:**

Freshwater duckweed, comprising the smallest, fastest growing and simplest macrophytes has various applications in agriculture, phytoremediation and energy production. *Lemna minor*, the so-called common duckweed, is a model system of these aquatic plants for ecotoxicological bioassays, genetic transformation tools and industrial applications. Given the ecotoxic relevance and high potential for biomass production, whole-genome information of this cosmopolitan duckweed is needed.

**Results:**

The 472 Mbp assembly of the *L. minor* genome (2*n* = 40; estimated 481 Mbp; 98.1 %) contains 22,382 protein-coding genes and 61.5 % repetitive sequences. The repeat content explains 94.5 % of the genome size difference in comparison with the greater duckweed, *Spirodela polyrhiza* (2*n* = 40; 158 Mbp; 19,623 protein-coding genes; and 15.79 % repetitive sequences). Comparison of proteins from other monocot plants, protein ortholog identification, OrthoMCL, suggests 1356 duckweed-specific groups (3367 proteins, 15.0 % total *L. minor* proteins) and 795 *Lemna*-specific groups (2897 proteins, 12.9 % total *L. minor* proteins). Interestingly, proteins involved in biosynthetic processes in response to various stimuli and hydrolase activities are enriched in the *Lemna* proteome in comparison with the *Spirodela* proteome.

**Conclusions:**

The genome sequence and annotation of *L. minor* protein-coding genes provide new insights in biological understanding and biomass production applications of *Lemna* species.

**Electronic supplementary material:**

The online version of this article (doi:10.1186/s13068-015-0381-1) contains supplementary material, which is available to authorized users.

## Background

Duckweed species comprise a group of aquatic monocotyledons macrophytes consisting of floating plant bodies or “fronds.” The family *Lemnaceae* consists of five genera, *Landoltia*, *Lemna*, *Spirodela*, *Wolffia* and *Wolffiella* among which 37 species have been identified so far [1–3]. Frond as well as root structures of duckweed have been morphologically simplified likely by natural selection to only those necessary to survive as floating aquatic plants. Duckweed species are of ecological significance as they are primary producers being a source of food for waterfowl, fish and small invertebrates and provide habitat for a number of small organisms. They are adapted to a wide variety of climatic regions where they, under favorable conditions, can grow extremely rapidly predominantly via asexual reproduction [4]. Such a vegetative growth results in genetically uniform clones, thereby eliminating potential effects due to genetic variability through meiosis. The natural characteristics of this plant family are mainly the basis why duckweed species are attractive for economic applications: duckweeds have been used as feed resource for fish, poultry, cattle’s and other animals [5, 6] and are utilized for wastewater treatment [7, 8]. Nowadays, duckweeds are genetically modified to improve industrial applications that seem to have great potential for bioenergy production [9, 10] and pharmaceutical applications [8, 11]. Recent work on duckweed species was highlighted in a special issue in Plant Biology to commemorate Dr. Elias Landolt and his contribution to modern duckweed research [12–14].

**Fig. 1:**
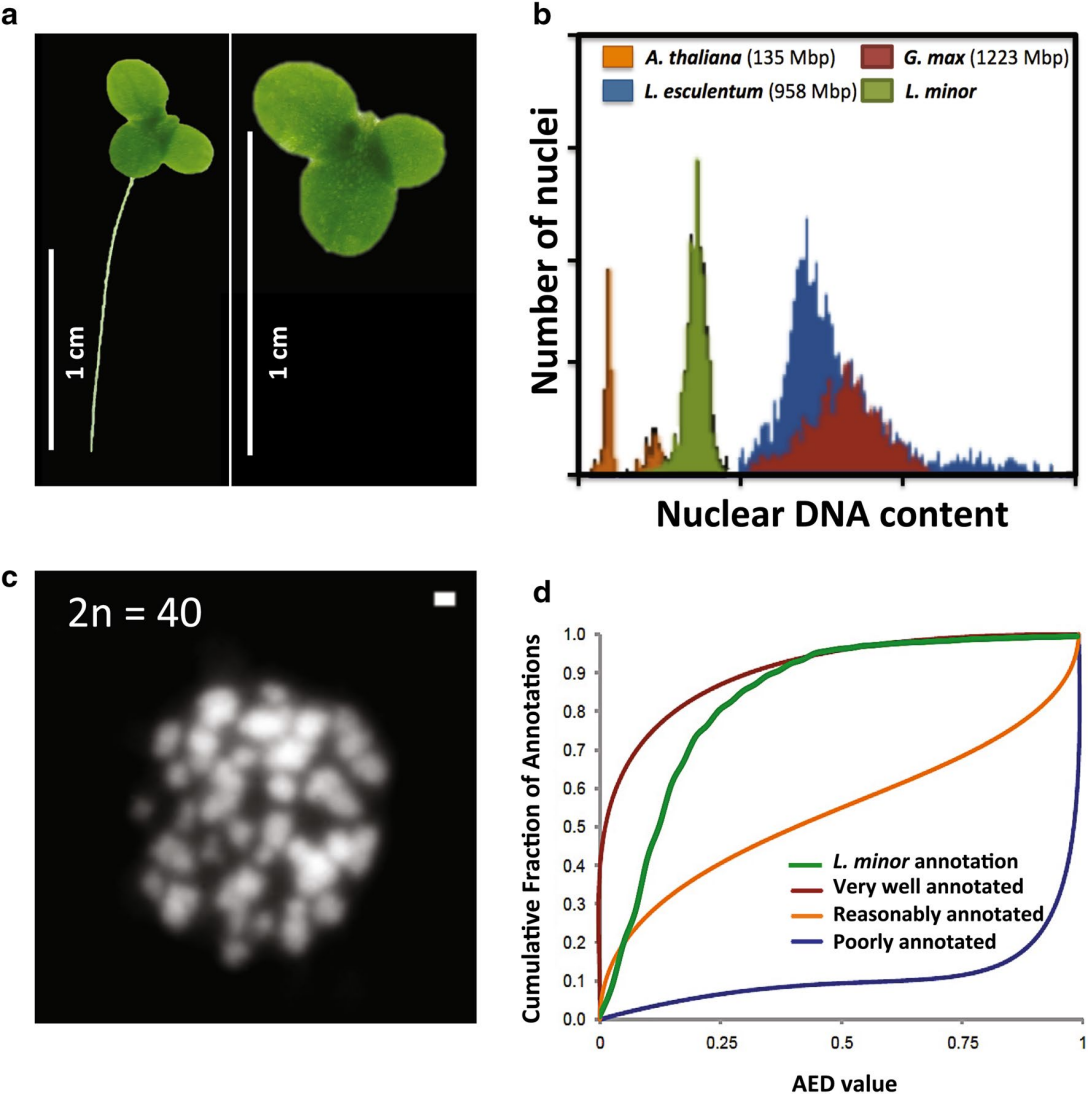
**a** Illustration of *L. minor* clone 5500. **b** Histogram of the relative DNA content from *L. minor* obtained by flow cytometry. A genome content of 481 Mbp was determined for *L. minor* genome using the DNA content of different plant species (Arabidopsis, tomato and soybean) as internal standard. **c** Picture of *L. minor* chromosomes of *L. minor* (2*n* = 40). The number of chromosomes was determined by DAPI staining. *Scale bar* 1 µm. **d** Evaluation of the *L. minor* genome annotation with AED annotation scoring value’s. The *L. minor* genome annotation is compared to reference curves from Maker-P annotation tool

Of all the duckweed species, Lemna species are probably the best known because of their extensive use in lab-based tests. Lemna species are smaller than *Spirodela* and *Landoltia* facilitating experimentation under limited spatial conditions but large enough to observe morphologic alterations without use of a microscope. Therefore, *L. minor* (Fig. 1a) was put forward as a model system to study fundamental plant research and has been shown to contribute to the understanding of the photoperiodic control of flowering [15, 16] and the discovery of auxin biosynthesis and sulfur assimilation pathways [17, 18]. Besides, their variety in growth habitats and their sensitivity to toxicants allowed the use of Lemna species in ecotoxicological research as representative of higher aquatic plants and standardized guidelines on how to perform a growth inhibition test were developed [19–26]. Testing procedures can follow the ASTM [24], ISO:20079 [26], OECD [19], Environment Canada [25], or EPA [23] test method guidelines. It is clear that duckweeds, and more in particular Lemna species, are gaining importance as physiological and ecotoxicological model organisms [27]. Survival, growth and reproduction at individual organisms are commonly used end points in ecotoxicity tests to support current risk assessments. However, as these end points are regarded as stringent, analyses at molecular level could provide a broader understanding in the alteration of biological pathways, including responses induced from environmental stressors [28]. Therefore, studies focusing on physiological mechanisms and genetic improvements among duckweed species await support at molecular level [3, 29].

The chloroplast genome of *L. minor* has been sequenced for phylogenetic analyses among other monocot plants [30]. Later on, Wang and Messing started with DNA barcoding duckweed strains [31] and sequenced chloroplast DNA from three other duckweed species [32], which allowed a taxonomic comparison between different duckweed ecotypes since their minute size, simple anatomy and the lack of flowering make it very harsh to analyze systematic relationships purely on morphological characteristics. To obtain nuclear DNA information for *Lemnaceae*, a whole-genome high-throughput sequencing project started in 2009 that aimed at sequencing the genome of the giant duckweed, *Spirodela polyrhiza*. Spirodela was chosen for its basal taxonomic position in the *Lemnaceae* and because of its small genome size of 158 Mbp, which is similar to that of Arabidopsis. The comprehensive genomic study of *S. polyrhiza*, published in 2014, provided insights into how this plant is adapted to rapid growth and an aquatic lifestyle [33]. Furthermore, physical mapping of the *S. polyrhiza* genome revealed that its 20 chromosomes are likely derived from seven ancestral chromosome blocks after two successive rounds of whole-genome duplication events [34].

In an effort to strengthen duckweed research, we employed the Illumina high-throughput sequencing technology to construct a draft genome of *L. minor* (Fig. 1a). Except for a small number of nuclear genes from small-scale individual studies [35], DNA information for *L. minor* is today limited to its chloroplast DNA [30]. An extensive analysis of genome sizes in different *L. minor* strains revealed that genome content and composition is highly variable within these species [36]. The estimated size of the haploid genome for a set of tested *L. minor* plants varied between 323 and 760 Mbp, thus two to three times larger than the genome size of *S. polyrhiza.* The primary objective was to characterize the protein-coding genes for *L. minor* clone 5500, a Lemna strain widely used in ecotoxicological research [37–42]. This genomic platform is expected to support the molecular basis of fundamental research in, e.g., ecotoxicogenomics and to facilitate the genetic improvement of this economically important plant, especially in biomass production.

## Results and discussion

### De novo assembly of *L. minor* genome with greater 100× of Illumina coverage

Genome of *L. minor* clone 5500 was estimated as 481 Mbp by flow cytometry (Fig. 1b) and is compacted in 20 chromosome pairs (2*n* = 40, Fig. 1c). In order to obtain the reference sequence of the *L. minor* genome, total genomic DNA was isolated to create two paired-end libraries for Illumina platform. A high-coverage 2 × 100 HiSeq library was supplemented with longer reads from a 2 × 300 MiSeq library. No gaps were included between both ends of the fragments resulting in paired-end reads having a nominal fragment length of 200 and 600 bp, respectively. HiSeq library consisted of 215,721,669 reads (43 Gbp) representing approximately a 90× genome coverage, while the Miseq library contained 26,270,063 (15 Gbp) reads equivalent to a genome coverage of 30×. After removing adaptors and reads containing unknown or low-quality nucleotides, the remaining 207,985,822 and 24,416,556 high-quality reads (coverage of 87× and 29× respectively) were used to assemble the *L. minor* genome (Additional file 1: Table S1). To obtain the best possible draft sequence, three different assembly programs were evaluated for the de novo assembly namely SOAPdenovo2 [43] and CLC bio, both using a *de Bruijn* graph-based algorithm and MaSuRCA [44] that uses an overlap-based assembly algorithm for the so-called super-reads. Such super-reads are uniquely extended short reads from high-coverage paired-end reads to significantly compress the data. Subsequently, the obtained assemblies were further processed with SSPACE [45] to scaffold, and Gapcloser [43] to close the gaps in a final step. With respect to number of contigs/scaffolds, corresponding N50 values and mismatch error frequency, it was found that draft genome generated by MaSuRCA generated a more robust genome sequence compared to the genomes generated by SOAPdenovo2 and CLC bio (Additional file 2: Table S2). MaSuRCA’s error-correction and super-reads processes reduced the raw paired-end reads to 2,145,090 super-reads that were applied to compute pairwise overlap between these reads. From these super-reads, the MaSuRCA pipeline generated 49,027 contigs (N50 contig size 20.9 kbp) and 46,105 scaffolds (N50 scaffold size 23.6 kbp) with a minimum length of 1000 bp (Additional file 2: Table S2). Therefore, scaffolds resulted from MaSuRCA were used for further downstream analysis.

Using the CEGMA pipeline [46], 233 protein-coding genes (94 %) of a set of highly conserved eukaryotic genes (248) were recognized within the MaSuRCA assembled genome of which 215 genes (86 %) were completely (>70 % of their length) covered (Additional file 3: Table S3). To assess the accuracy of the de novo assembly, a de novo generated set of transcripts coming from the same *L. minor* strain was aligned to the scaffolds. Using BLAT software [47], it was found that ~97 % of the cleaned transcripts aligned to at least one scaffold, with ≥95 % coverage and ≥90 % sequence identity (Additional file 4: Table S4). The final assembled sequence spanned 472,128,703 bases embedded in 46,047 scaffolds, with an N50 length of 23,801 bases when scaffolds of 1000 bp or smaller are excluded. This length is similar to the predicted genome size using Kmergenie [48] that estimated the assembly size to 475 Mbp based on k-mer statistics, or to 481 Mbp using flow cytometry (Fig. 1b). Therefore, as a proportion of the nuclear DNA content, the *L. minor* genome sequence was almost fully (98.15 %) covered by the assembled scaffolds. Scaffolds having a sequence length of 2 kbp or more covered about 96 % in size of the de novo genome assembly sequence of which 17 scaffolds had a minimum sequence length of 0.5 Mbp (Additional file 5: Figure S1). Using the available *L. minor* chloroplast DNA data, the full chloroplast genome of *L. minor* clone 5500 was obtained here by aligning NGS reads using BWA with Genbank *L. minor* chloroplast genome as reference (NC_010109.1) [49]. This chloroplast genome was 165.9 Mbp and contained 48 variants related to 117 bp (0.07 %) compared to the Genbank reference sequence which is originally from a different clone/ecotype (Additional file 6: Table S5).

**Fig. 2:**
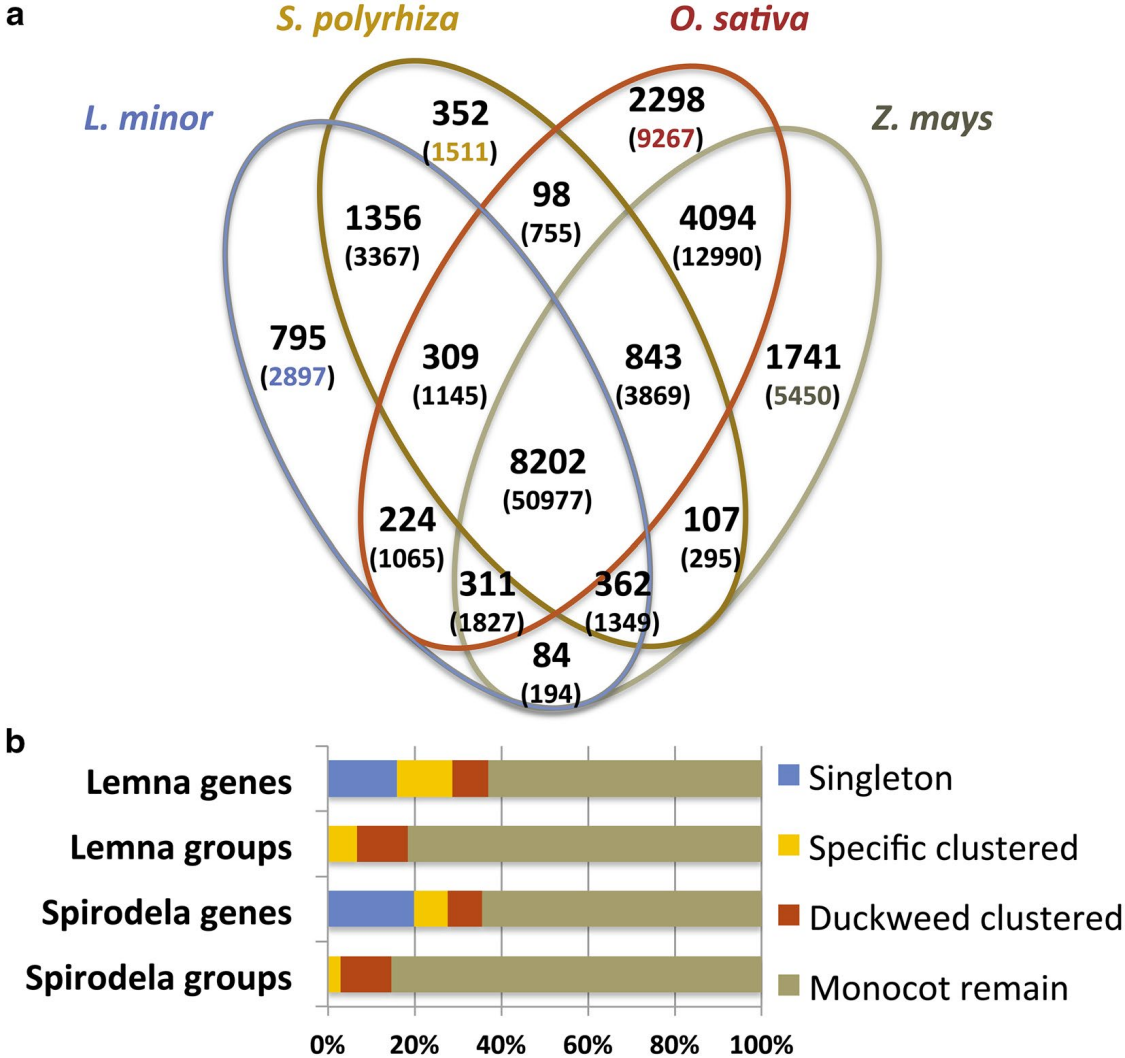
A venn diagram showing clusters of orthologous and paralogous gene families in *L. minor*, *S. polyrhiza*, *Z. mays* and *O. sativa* as identified by OrthoMCL. Gene family number is listed in each of the components; the number of genes within the families for all of the species within the component is noted within parentheses

In this study, a whole-genome shotgun approach was used to sequence *L. minor* genome using de novo assembly of exclusively paired-end read libraries which resulted in a moderate N50 value. The lack of mate-pair libraries makes a significant difference in the size of scaffolds and thus also to the N50 value. Libraries of paired-end reads simply cannot span many of the repetitive sequences in a genome, especially in plant genomes, which are known to have a high amount of repetitive sequences [50]. The involvement of a set of mate-pair libraries would produce longer scaffolds making N50 values 10–100 times higher [51]. Our genome assembly contains a scaffold N50 value of more than 20 kbp, which is comparable to the scaffold N50 value of the genome assemblies from *Cannabis sativa* [52] and *Phoenix dactylifera* [53]. Furthermore, the generated N50 values of other sequenced plant genome assemblies at which no mate-pair libraries are included (scaffold N50 value) are also in line to the here obtained scaffold N50 value [51]. This suggests that the produced *L. minor* assembly covers most of the non-repeated sequences. New sequencing libraries together with mapping information such as physical maps, optical maps, or cytogenetic maps [34] may be needed to improve the quality of genome sequence in order to analyze comparative genomics, whole-genome duplications, or genome evolution in duckweed species. However, current assembly enables us to characterize the basic elements (e.g., repeat and gene content) of the *L. minor* genome.

### Repetitive sequences comprise 62 % of the *L. minor* genome assembly

Homology-based comparisons revealed that 62 % of the *L. minor* genome assembly consisted of repetitive sequences (Table 1). The repeats were categorized in retrotransposons (31.20 %), DNA transposons (5.08 %), tandem repeats (3.91 %) and other unclassified repeats (21.27 %). Long terminal repeat (LTR) retrotransposons are the predominant class of transposable elements (29.57 %), which is consistent with other plant genomes.

**Table 1:** De novo identification of sequence repeats in the genome of *L. minor*

Class	Number of elements	Elements percentage (%)	Sequence occupied (bp)	Sequence percentage of transposable elements (%)	Sequence percentage of genome (%)
Retrotransposons	223,595	34.09	152,153,999	50.76	31.20
LTR *Copia*	124,171	18.93	91,641,466	30.57	18.79
LTR *Gypsy*	81,828	12.48	51,647,357	17.23	10.59
LTR other	4532	0.69	943,224	0.31	0.19
LINE	10,193	1.55	6,598,659	2.20	1.35
SINE	2871	0.44	1,323,293	0.44	0.27
DNA transposons	54,699	8.34	24,778,060	8.27	5.08
Tandem repeats	152,112	23.19	19,062,495	6.36	3.91
Satellite	7243	1.10	2,821,147	0.94	0.58
Low complexity	18,075	2.76	1,464,636	0.49	0.30
Simple repeats	126,794	19.33	14,776,712	4.93	3.03
Unclassified	225,397	34.37	103,759,727	34.61	21.27
Total	655,803	100	299,754,281	100	61.46

The most abundant transposon families were *gypsy* and *copia*, contributing to 10.59 and 18.79 % of the genome, respectively. For the DNA transposable elements, it was found that DNA_*hAT*-*Ac* elements were most abundant spanning almost 2.7 % of the nuclear genome. The high proportion of repetitive sequences could explain for the dispersed distribution of heterochromatin signatures of the *L. minor* clone 8623 (377 Mbp, [54]). Given that the plasticity of genome size in different *L. minor* clones (ranging from 323 to 760 Mbp) [36, 55] could result from different repetitive amplification and/or recent whole-genome duplications, it is interesting to study repeat content and karyotype of different *L. minor* geographical clones. In comparison with the *S. polyrhiza* genome [33] which is the most ancient duckweed, repeat amplification in *L. minor* could explain 94.5 % of the genome size difference between two duckweed reference genomes. Surprisingly, the LTR *copia* is more abundant than LTR *gypsy* in the *L. minor* genome. The *gypsy/copia* ratio in *L. minor* is 0.56, whereas the corresponding ratio in *S. polyrhiza* is 3.5 [33]. Although our repeat identification method is assembly dependent, implying the repeat content could be underestimated and high unclassified repeat proportion (34.37 % repeat content, Table 1), repeat content in *L. minor* suggests that the amplification of LTR retrotransposons played an important role in duckweed genome evolution. More detailed repeat characterization in published or ongoing duckweed genomes sequencing projects could shed more light on this interesting story.

### *L. minor* 5500 contains a similar number of protein-coding genes as *S. polyrhiza* 7498

Scaffolds of 2 kbp or longer were selected for gene prediction, as gene predictors require a certain amount of sequence upstream and downstream of a gene to work accurately. Therefore, scaffolds smaller than 2 kbp were skipped in order to reduce the false positive errors and fragmented gene models in gene prediction. The CEGMA tool was utilized to assess the completeness on this selection of scaffold sequences. It was found that still 213 full-length genes were completely aligned meaning that the final number of the gene annotation represents at least 85 % of the true number of genes (Additional file 3: Table S3). Gene models from masked *L. minor* genome sequences were predicted and annotated with the ab initio and homology-based gene prediction pipeline MAKER-P [56] (Additional file 7: Table S6). To obtain a comprehensive set of *L. minor* gene models, RNA was isolated and sequenced from *L. minor* plants cultivated under healthy growth conditions and from *L. minor* plants exposed to various stress conditions (including uranium, gamma radiation and Sr-90 treatment). Using the Illumina HiSeq platform, approximately, 592,326,402 clean sequencing reads were obtained after adapter and low-quality reads trimming (Additional file 8: Table S7). 530,159 transcripts were produced with Trinity de novo assembler, including different isoforms per transcript [57]. These transcriptomic data of *L. minor*, together with all available transcripts from duckweed species *Landoltia punctata*, *Lemna gibba* and *S. polyrhiza* and supplemented with nine proteomes from monocotyledon plants, served as evidence for the gene prediction tools SNAP [58] and Augustus [59] inside Maker-P pipeline. In total, 22,382 protein-coding genes were annotated whereof 18,744 genes (84 %) contained an AED (Annotation Edit Distance) score under 0.25 which can be regarded as highly accurate (Fig. 1d). Although the number of genes is lower than the number found in other sequenced monocot plants, it was very similar to that of the closely related *S. polyrhiza*. This supports the hypothesis that the small and structurally simple anatomy of duckweed species allowed to loose a number of genes. On average, gene models consisted of 1934 bp and means of 4.8 exons per gene (Table 2; Additional file 9: Figure S2). The exon length distribution was consistent with other species, although *L. minor* intron length tended to be shorter than that of other species used in the comparison (Table 2). To assess the accuracy of the obtained annotation, the complete set of the *L. minor* proteins from the National Center of Biotechnology Information (NCBI) was blasted to the *L. minor* proteins. It turned out that 60 of the 61 NCBI accessions (downloaded 11-09-2015) could be aligned to at least one of the *L. minor* proteins (BLASTP [60], e-value of 1e−10) (Additional file 10: Table S8).

**Table 2:** Overview of gene features from *L. minor* and three other monocotyledonous plants

Species	*L. minor*	*S. polyrhiza*	*O. sativa*	*Z. mays*
Genome size (Mbp)	481	158	430	2.067
No. Of genes	22,382	19,623	39,045	63,480
Mean gene length (bp)	2738	3458	2853	4653
Median gene length (bp)	1934	2245	1654	2397
Mean CDS length	1332	1108	1064	1206
Median CDS length	1146	903	849	996
Mean exon length	208	213	259	333
Median exon length	138	121	139	178
Mean exons per mRNA	4.8	5.2	4.9	5.5
Median exons per mRNA	3	4	3	4
Mean intron length	209	560	418	878
Median intron length	103	178	170	144

Since the *L. minor* genome has been sequenced using a WGS approach without the use of mate-pair libraries or the construction of a physical map, it is not excluded that some alleles may have been annotated as individual genes. Heterozygosity is namely more prevalent in asexual individuals compared to sexual species through mutation accumulation in clonal lineages [61]. A study of Cole and Voskuil revealed that this was also true for a population of *L. minor* [55]. However, when using the MaSuRCA pipeline instead of *de Bruijn* graph-based assembly approach, it overcomes the repeat sequences, errors, low-coverage regions and small structural differences caused by heterozygosity because of its overlap-layout-consensus approach [62]. To assess the accuracy of the de novo annotation, we examined the proportion of de novo created transcripts represented in the annotated transcriptome. A total of 179,736 different RNA transcripts were made by Transdecoder of which 179,734 could be mapped to the annotated transcripts (BLASTN [60], e-value of 1e−30).

### Lemna proteome is mostly (66.2 %) shared with the Spirodela proteome

To study gene content of *L. minor* and duckweed in general, we examined the sequence similarities between *L. minor* and *S. polyrhiza* genes and two other highly annotated monocot plants. Therefore, the 22,382 gene products of *L. minor* were clustered into orthologous and paralogous groups with 107,716 gene products from *S. polyrhiza*, *Oryza sativa* and *Zea mays* using OrthoMCL [63]. Although the three sets of gene annotation contain different numbers of gene models reflecting the different annotation history, this comparison provided an indication of the overall completeness of our assembly. In summary, 8202 orthologous groups were conserved in all four species containing 39 % of the submitted genes (Fig. 2a). In addition to 3546 *L. minor* singleton genes (not grouped by OrthoMCL, 15.8 % of total *L. minor* genes), a total of 795 paralogous groups representing 2897 genes (12.9 %) were unique to *L. minor* (Additional file 11: Table S9). These 6443 genes from two groups are further referred to as Lemna-specific genes in this study. The more closely related species would be expected to have a higher numbers of similar gene models. As a result, 14,830 *L. minor* genes (66.2 %) have orthologs in *S. polyrhiza,* whereas other 1109 *L. minor* genes (4.9 %) have orthologs in either *O. sativa*, *Z. mays,* or both but not *S. polyrhiza* (Fig. 2b). Furthermore, it was found that 1821 genes (8.13 %) of *L. minor* shared a unique similarity with at least one gene from *S. polyrhiza*, which are further referred to as duckweed-specific genes.

It has been shown in the *S. polyrhiza* genome that there have been two ancient rounds of whole-genome duplications during evolution (ca. 90 Mya) [33]. In the comparison of gene families between *S. polyrhiza* and four representative plant species (Arabidopsis, tomato, banana and rice), a low gene copy number in *S. polyrhiza* indicated preferred gene losses of duplicated genes [33]. It would be interesting to study the gene number and relation of gene families of other Lemna genomes which are in progress, such as *L. gibba* G3 DWC131 (450 Mbp) and *Lemna minor* clone 8627 (800 Mbp) [64, 65]. It is conceivable that the ancestor genome of Lemna species contained at least one recent whole-genome duplication after the split between *L. minor* and *S. polyrhiza* genera followed by different degree of gene removal processes of duplicated genes resulting to different Lemna species with the genome size ranging from 323 to 760 Mbp [36, 55]. The most extensive gene loss can result to a reduced total gene numbers such as the case of *L. minor* 5500. An alternative hypothesis, on the other hand, could be that *L. minor* 5500 represents the Lemna ancestor genome which contains the similar gene content as the Spirodela genome. Other larger genome Lemna species could have been evolved from larger repeat expansion or very recent and independent whole-genome duplications. This hypothesis could be tested by future work, which studies macro-synteny relationship between *S. polyrhiza* 7498 genome (2*n* = 40, 158 Mbp) and *L. minor* 5500 genome (2*n* = 40, 481 Mbp).

### Gene annotation information supports further genome functional analysis and biomass production applications

**Fig. 3:**
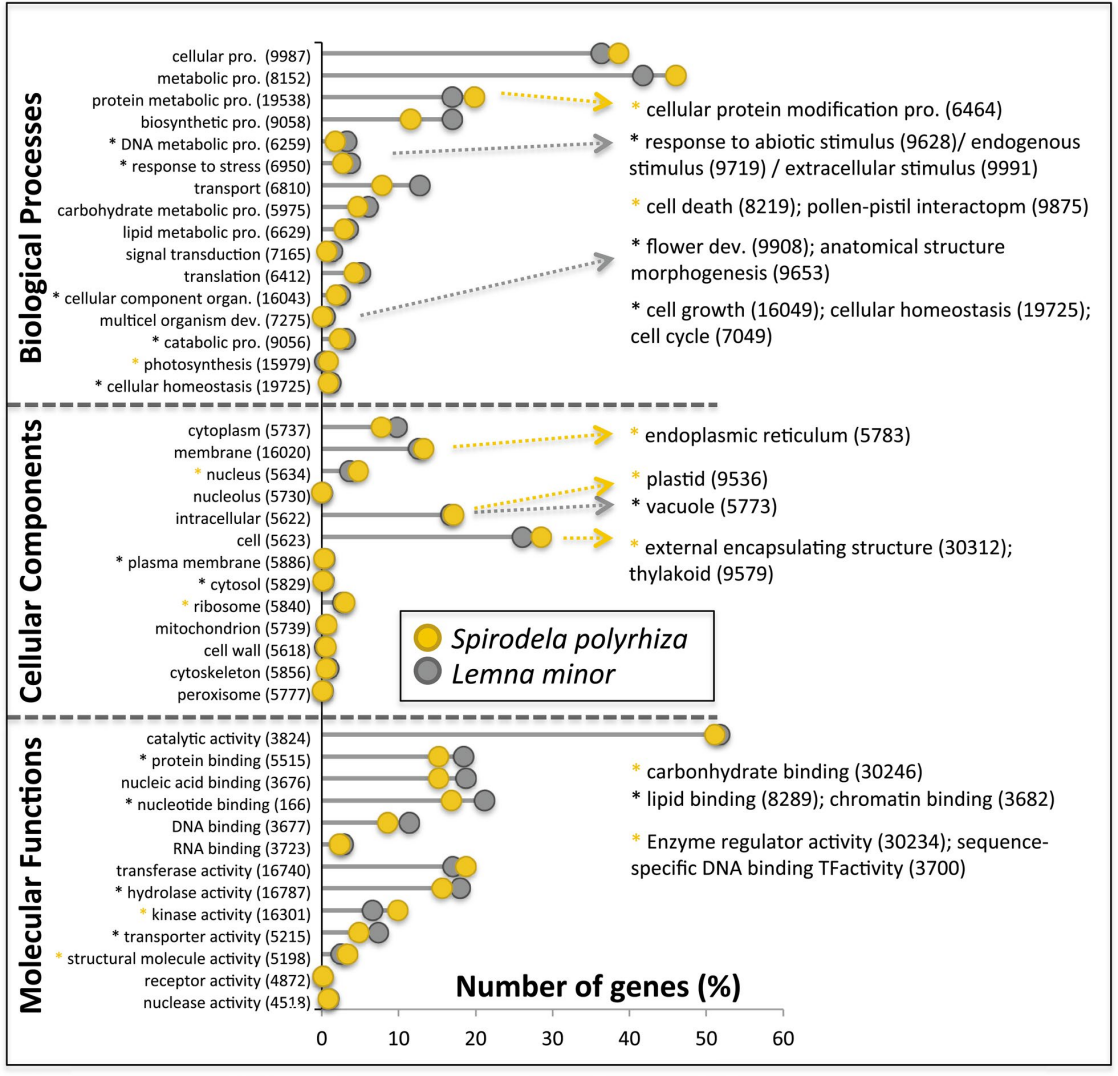
Comparison of the most relevant plant GO slim terms for three structured ontologies between *L. minor* (*black*) and *S. polyrhiza* (*yellow*). More specific GO terms over over/under represented in *L. minor* are shown on the right side. *Asterisk symbols* indicate that these GO terms are significantly enriched (Fisher exact test, FDR <0.05) in *L. minor* (*black*) or *S. polyrhiza* (*yellow*) (Fisher exact test, FDR <0.05). *pro* process, *organ.* organization, *dev.* development, *TF* transcriptional factor

To identify the putative functions of the *L. minor* gene models, a sequence similarity search was carried out against the Swiss-Prot protein sequences of *Arabidopsis thaliana* and *O. sativa* (BLASTP [60], e-value of 1e−5). Subsequently, the transcripts were annotated with Gene Ontology (GO) and Pfam terms using a local installation of Interproscan 5 [66] and KEGG pathway mapping using the KEGG Automatic Annotation Server (KAAS) [67]. The pfam-A database provides profile hidden Markov models of over 13,672 conserved protein families [68]. The GO project provides an ontology of defined terms representing gene product properties, which covers three domains: cellular component, molecular function and biological process. The result of KAAS contains KO (KEGG Orthology) assignments and automatically generated KEGG pathways. In total, 21,263 gene models (95 %) received an annotation link with at least one of the included databases of which 18,597 (83.1 %) were assigned to one or more Pfam domains, 7329 (32.7 %) to KEGG ontology term and 15,512 (69.3 %) of the proteins were successfully annotated with Gene Ontology terms. The GO terms of *L. minor* present overall similarity to the GO annotations of *S. polyrhiza*, *O. sativa* and *Z. mays* (Fig. 3, Additional file 12: Figure S3; Additional file 13: Table S10). The GO enrichment analysis between the two duckweed species reveals that the *L. minor* proteome contains 24 overrepresented and 15 underrepresented GO terms with significant FDR <0.05 (Fig. 3; Additional file 14: Table S11). Enriched proteins in *L. minor* 5500 included (1) enzymes involved in catabolic processes (GO:9056, 422 proteins), hydrolase activity (GO:16787, 2739 proteins); (2) proteins in response to various stimulus (e.g., stress (GO:6950, 529 proteins), abiotic stimulus (GO:9628, 86 proteins), extracellular stimulus (GO:9991, 19 proteins), endogenous stimulus (GO:9719, 55 proteins); and (3) biosynthesis processes (e.g., precursor metabolites and energy (GO:6091, 258 proteins), DNA metabolic process (GO:6259, 350 proteins), carbohydrate metabolic process (GO:5975, 776 proteins). These proteins could contribute to *L. minor* ability for (1) the removal of surplus nutrients from waste water, (2) adaptation to various climate conditions resulting in their world-wide distribution, and (3) providing nutritional value and high biomass productivity. Interestingly, 2381 *L. minor* specific genes (36.9 %) and 326 *L. minor* tandem duplicated genes (17.4 %) are present in the overrepresented GO terms. Furthermore, *L. minor* contains sequences coding for 12 glutamine synthetases (GS) and 21 glutamate synthases (GOGAT) in comparison with 7 and 11 sequences in *S. polyrhiza*, respectively (Additional files 15, 16: Fig. S4, S5; Additional file 17: Table S12). Both enzymes regulate ammonium assimilation which is an important biochemical pathway for the use of *L. minor* in wastewater remediation, possibly in combination with energy production [69]. Therefore, these amplified genes, which may diverge to produce novel functions via neofunctionalization, could be potential candidates for further functional studies, since efficient transformation protocols for *L. minor* are available [70, 71].

## Conclusions

In the present study, the *L. minor* genome has been sequenced using exclusively paired-end sequencing reads. Given an estimated genome size of 481 Mbp, the draft genome represented 98 % of the *L. minor* genome and contained protein-coding genes proportional to *S. polyrhiza*. Functional characterization and comparison supported the accuracy of the obtained predicted protein-coding genes. Therefore, the present *L. minor* genomic resource is highly beneficial for understanding the biological and molecular mechanisms in *L. minor* and will facilitate future genetically improvements and biomass production applications of duckweed species.

## Methods

### Plant material

*Lemna minor* cv. Blarney plants (Serial number 1007, ID number 5500) were collected from a pond in Blarney, Co. Cork, Ireland, (University College Cork, Ireland) and are a gift from Prof. Dr. M. Jansen. The plants were aseptically cultured in a growth chamber in 250-mL glass Erlenmeyer flasks containing half-strength Hütner medium [27] under continuous light (Osram 400 W HQI-BT daylight, OSRAM GmbH, Augsburg, Germany, 102 ± 1 µmol m^−2^ s^−1^) at 24.0 ± 0.5 °C. Plants were subcultured every 10–12 days by transferring three plants to 100 mL of fresh growth medium.

### DNA extraction and genome assembly

Genomic DNA was extracted from fresh *L. minor* plants with the DNAeasy Plant kit mini prep (Qiagen, Venlo, Limburg, Netherlands) according to the manufacturer’s recommendations. Aliquots of the extraced DNA were used for measuring DNA quantity with NanoDrop ND1000 and for genome sequencing.

We sequenced the *L. minor* genome by exclusively using Illumina platforms which were performed at VIB Nucleomics Core, Leuven, Belgium. Two sequencing runs were performed: A 2X100 paired-end library was used to generate a high-coverage library using the HiSeq 2000 platform, while a 2X300 paired-end library was used for the MiSeq platform (Additional file 1: Table S1). The produced reads were cleaned using the FastX tool [72] and cutadapt [73]. High-quality paired-end sequence reads were used as input for three different assembly programs: SOAPdenvo2 [43], CLC Bio (genomic workbench 7-Qiagen, Aarhus, Denmark), and MaSuRCA pipeline [44]. For SOAPdenovo2 and CLC Bio, overlapping paired-end reads were first merged before assembly using Flash (1.2.8). A k-mer length of 63 was selected for both SOAPdenovo2 and CLC Bio. Using the software package MaSuRCA pipeline, a K-mer length of 81 has been selected by jellyfish, included in the MaSuRCa pipeline, which was close to the k-mer length of 87 suggested by Kmergenie. Since this pipeline generates super-reads, raw sequencing reads were used as input data. The scaffolds/contigs resulted from three different assemblers were further processed with SSPACE [45] to scaffold the contigs, and Gapcloser [43] to close the gaps in a final step. Afterwards, The 46,047 scaffolds that could be mapped with >95 % of their sequence length to the *L. minor* chloroplast genome were excluded from the scaffold pool resulting in 45,990 scaffolds. The chloroplast genome was assembled using BWA with genbank *L. minor* chloroplast sequence as reference (NC_010109.1). GATK tools were used for variant calling.

### Accession numbers and availability of material

All the raw sequence data of the *L. minor* genome and transcriptome have been deposited at the NCBI Sequence Read Archive (SRA) repository under the SRA accession number SRP065561. The *L. minor* genome with annotation is available at CoGe database with the genome identifier ID 27408 (https://genomevolution.org/r/ik6h) or upon request (avhoeck@gmail.com; nhoreman@sckcen.be).

### Annotation of repeat sequences

Putative transposable elements of the *L. minor* genome assembly were identified using REPEATMASKER version 3.3.0 (http://www.repeat-masker.org) using Dfam library (Dfam 1.3), RepBase library (13 July 2014) and a de novo *L. minor* library. RepeatModeler was used to build a *L. minor* de novo repeat library.

### RNA extraction and de novo assembly

RNA was extracted from plants exposed to different concentrations of uranium-238, gamma radiation, and strontium-90. For the gamma radiation treatment, plants were exposed to dose rates of 15, 53, 120, 232 mGy h^−1^ using a cesium-137 gamma source (1.25E + 12 Bq) in a modified OECD medium [42]. For the beta-radiation treatment [41], plants were cultured in a modified growth medium containing ^90^Sr activity concentrations of 25, 250, 2500, and 25,000 kBq L^−1^ added as SrCl_2_ (3.7 MBq stock solution, IDB Belgium), while for uranium treatment [37], plants were exposed to a growth medium containing uranium concentrations of 0.5, 4, 6.5, and 10 µM added as UO_2_(NO_3_)_2_ H_2_O. For each treatment, control plants were grown simultaneously under non-exposure conditions. After 7 days of exposure, plants from each exposure condition (3 replicas) were snap frozen in liquid nitrogen and stored at −80 °C until total RNA extraction. Total RNA was extracted from *L. minor* plants with RNeasy Plant Mini Kit (Qiagen, Venlo, Limburg, Netherlands) according to the manufacturer’s recommendations. RNA quality and quantity control was performed using NanoDrop ND1000 and BioAnalyzer (Agilent Technologies).

Transcriptomes of full *L. minor* plants were sequenced on the Illumina HiSeq 2000 platform at the University of Antwerp using the Truseq™ RNA sample prep kit (version 2—single read—50 bp) according to the manufacturer’s recommendation. The reads were cleaned using FASTX-Toolkit [72] and cutadapt [73]. The reads of all exposure concentration per stress condition (five different concentrations) and of control plants were pooled and served as input for the de novo transcriptome assembly program Trinity [57]. TransDecoder [50] was used to identify likely coding sequences within Trinity generated transcript sequences after BLASTP (threshold 1e−5) selection with Swiss-Prot database [74]. The obtained Transcdecoder_transcripts.fasta file was used within the gene prediction tool to serve as transcript evidence.

### Gene predictions

Gene prediction for *L. minor* was conducted by various methods available in MAKER-P version 2.31.8 [56]. The MAKER-P annotation pipeline consists of different steps to generate high-quality annotations by taking transcriptomic as well as proteomic evidence. Only scaffolds with a minimum sequence size of 2000 bp were considered for gene prediction. Hence, the 32,348 scaffolds were masked with RepeatMasker using the same libraries as described above. MAKER-P was run on *L. minor* using assembled transcriptomic data (Transcdecoder_transcripts.fasta), and proteins from monocotyl plants (*Musa acuminata*, *Musa balbisiana*, *Sorghum bicolor*, *O. sativa* subsp. *japonica*, *Brachypodium distachyon*, *Elaeis guineensis*, *S. polyrhiza* and *P. dactylifera* (downloaded february 17, 2014 from Swiss-Prot database). The gene prediction tools SNAP [58] and Augustus (3.0.1) [59] were trained to generate species-specific gene models. The first run for initial prediction was made by de novo assembled Transcdecoder transcripts data. The output results were used to retrain SNAP inside MAKER-P pipeline using both assembled transcdecoder transcripts and protein evidence. The resulting second gene set was used for the final training of SNAP. Augustus was trained using the output of CEGMA with WebAugustus. An ad hoc filtering procedure was applied to the genes consisting of one exon as single-exon alignments often result from spurious alignments, library contamination, background transcription of the genome, pseudogenes, and repeat elements [56]. Therefore, the proteins of the single-exon gene models that did not supported on transcript level were aligned to the proteins from the monocot plants included in the gene prediction tool. Single-exon genes were retained when its protein length is 90 % or more compared to at least one protein from the monocot plants (BLASTP, e-value of 1e−5).

### Functional annotation and GO enrichment analysis

Putative gene function was assigned to the *L. minor* genes based on the best alignment to the protein sequences of Swiss-prot using BLASTP [35] with a threshold of 1e−5. Gene ontology terms and Pfam domains were assigned to the genes by software Interproscan 5 [66]. GO slim identification and representation of the GO terms were performed using BINGO [75]. Two-sided Fisher’s exact test with false discovery rate (FDR) threshold of <0.05 was used for enrichment analysis in Blast2GO v3.1.3 [76]. The enriched GO terms were further filtered to reduce to the most specific GO terms.

### Identification of orthologous groups and tandem duplicated genes

Proteins of *L. minor*, *S. polyrhiza*, *O. sativa*, and *Z. mays* were selected to perform an all-against-all comparison using BLASTP [35]. The results were fed into the stand-alone OhrthoMCL [63] program using a default MCL inflation parameter of 1.5. Tandem duplicated genes were detected in 10 kb or longer scaffolds of *L. minor* by using SynMap tool of CoGe website [77]. Cladogram analyses were performed using default parameters using advanced workflow of phylogeny.fr [78].

### Flow cytometry and chromosome counting

Flow cytometry was used to experimentally estimate DNA genome size of *L. minor*, by comparing it to the DNA content of *Lycopersicon esculentum*, *Glycine max,* and *Arabidopsis thaliana*. The isolation of nuclei was performed on fresh plant material (fronds) immediately after harvesting using the Cystain PI Absolute P kit (Partec). *L. minor* fronds were chopped with a fresh razor blade in a petri dish containing 250 µl ice-cold extraction buffer. After 1 min incubation, the solution was filtered through a 50-μm nylon filter (Celltrics), and 1 mL staining solution, consisting of 1 mL staining buffer, 120 μL propidium iodide (PI) solution, and 6 μL RNase per sample, was added to the flow-through. The samples were then incubated in the dark for at least 1 h, and their nuclear DNA content was analyzed on the BD Accuri C6 Flow Cytometer (BD Biosciences) with a FL2 585/40 nm filter. Reference DNA standards suitable for plant genome size estimation were received from the lab of J. Dolezel. At least, 500 nuclei counts per plant were analyzed.

Metaphase chromosome preparation of *L. minor* clone 5500 was performed as described in [34] with a small modification. Root tips which were macerated in a drop of 75 % acetic acid were treated further with nine acetic acid:one methanol solution for 3 min before squashing. Chromosome counting was evaluated by DAPI staining (2 µg mL^−1^ in VectaShield) by using a 100× objective of a Zeiss Axioplan 2 epifluorescence microscope equipped with a cooled CCD camera (Diagnostic Instruments, Inc.).

## Additional files

10.1186/s13068-015-0381-1 Illumina libraries statistics for genome assembly.
10.1186/s13068-015-0381-1 Summary statistics of the *L. minor* genome assemblies.
10.1186/s13068-015-0381-1 Number of CEGMA hits for different genome assemblies.
10.1186/s13068-015-0381-1 Statistics of transcriptome and whole genome assembly of *L. minor.*.
10.1186/s13068-015-0381-1 Assembled scaffold length distribution of *L. minor* genome.
10.1186/s13068-015-0381-1 Variant calling of *L. minor* (strain 5500) chloroplast genome vs. *L. minor* GenBank reference genome NC_01019.
10.1186/s13068-015-0381-1 Summary of the masked and unmasked genome assembly.
10.1186/s13068-015-0381-1 Illumina libraries statistics for transcriptome assembly.
10.1186/s13068-015-0381-1 Distribution of the number of exons per gene.
10.1186/s13068-015-0381-1 BLAST results of* L. minor* GenBank genes vs* L. minor* 5500.
10.1186/s13068-015-0381-1 Overview of OrthoMCL gene clusters for* L. minor* with* S. polyrhiza*,* Z. mays* and* O. sativa*.
10.1186/s13068-015-0381-1 Distribution of top plant GO slim categories for *L. minor*, *S. polyrhiza*, *Z. mays*, and *O. sative* proteomes. GO slim categories were assigned to *L. minor* by InterProscan5. GO slim categories of *S. polyrhiza* were extracted from Phytozome10 and *O. sativa* and *Z. mays* from Plaza 3.0.
10.1186/s13068-015-0381-1 Overview of gene ontology classification.
10.1186/s13068-015-0381-1 Overview of the over and underrespresented GO terms for *L. minor* genes compared to S. polyrhiza genes and for L. minor and duckweed specific genes in *L. minor* genome. The number of tandem genes for each GO term are also included.
10.1186/s13068-015-0381-1 Cladogram of glutamate synthase isoforms in *L. minor* (Lminor), *S. polyrhiza* (Spipo), *A. thaliana* (AT), O. indica (OSINDICA) and *B. distachyon* (BD). The phylogentic relationships of glutamate synthase from different species have been calculated under default parameters using phylogeny.fr.
10.1186/s13068-015-0381-1 Cladogram of glutamine synthetase isoform in *L. minor* (Lminor), *S. polyrhiza* (Spipo), *A. thaliana* (AT), O. indica (OSINDICA) and *B. distachyon* (BD). The phylogentic relationships of glutamine synthetase from different species have been calculated under default parameters using phylogeny.fr.
10.1186/s13068-015-0381-1 Blast hit results of *L. minor* glutamine synthetase and glutamate synthase genes.

## Abbreviations

ASTM: American Society for Testing and Materials
OECD: Organisation for Economic Co-operation and Development
EPA: Environmental Protection Agency
LTR: long terminal repeats
AED: annotation edit distance
GO: gene ontology
KEGG: kyoto encyclopedia of genes and genomes
