# Supplementary material for: The first draft genome of the aquatic model plant Lemna minor opens the route for future stress physiology research and biotechnological applications
Source: Biotechnol Biofuels. 2015 Nov 25;8:188. doi: 10.1186/s13068-015-0381-1 (PMC4659200; doi:10.1186/s13068-015-0381-1)
Supplement: Supplementary file 1 — 10.1186/s13068-015-0381-1 Illumina libraries statistics for genome assembly. [file 13068_2015_381_MOESM1_ESM.docx]

**Supplementary Table S1:** Illumina libraries statistics for genome assembly

| Organism: Lemna minor | Organism: Lemna minor |
| --- | --- |
| Platform: HiSeq | Platform: MiSeq |
| Library Prep Kit: TruSeq | Library Prep Kit: TruSeq |
| Sequencing Kit: TruSeq Reagent Kit (200-cycles) | Sequencing Kit: MiSeq Reagent Kit v3 (600-cycles) |
| fragment: paired end | fragment: paired end |
| mean insert size = 566 | mean insert size = 199 |

| Yield statistics raw reads Hiseq | | | |
| --- | --- | --- | --- |
|  | # Bases | Fragments | Read Length |
|  | 43.575.777.138 | 215.721.669 | 101 X 2 |
| Yield statistics processed reads | | | |
|  | 40.730.561.447 | 207.985.822 | 98,60 X 2 |
|  |  |  |  |
| Yield statistics raw reads Miseq | | | |
|  | # Bases | Fragments | Read Length |
|  | 15.386.811.234 | 26.270.063 | 297,38 X 2 |
| Yield statistics processed reads | | | |
|  | 12.861.776.775 | 222.080.278 | 287,74 X 2 |
