# Supplementary material for: The first draft genome of the aquatic model plant Lemna minor opens the route for future stress physiology research and biotechnological applications
Source: Biotechnol Biofuels. 2015 Nov 25;8:188. doi: 10.1186/s13068-015-0381-1 (PMC4659200; doi:10.1186/s13068-015-0381-1)
Supplement: Supplementary file 2 — 10.1186/s13068-015-0381-1 Summary statistics of the L. minor genome assemblies. [file 13068_2015_381_MOESM2_ESM.docx]

**Supplementary Table S2:** Summary statistics of the *L. minor* genome assemblies

|  |  |  |  |  |  |  |  |  |  |  |  |
| --- | --- | --- | --- | --- | --- | --- | --- | --- | --- | --- | --- |
|  |  | **Masurca** | | | | **SOAPdenovo** | | | **CLCbio** | | |
| **Assembly** |  | **contigs** | **scaffolds** | **SSPACE** | **gapclose** | **scaffolds** | **SSPACE** | **gapclose** | **scaffolds** | **SSPACE** | **gapclose** |
| # scaffolds |  | 49027 | 46105 | 46049 | 46047 | 64259 | 64257 | 64226 | 72538 | 72473 | 72479 |
| Total length |  | 470870040 | 472027693 | 472134576 | 472128703 | 370889226 | 371565543 | 369291063 | 437943651 | 438020551 | 438098258 |
| Largest contig |  | 1663208 | 1663208 | 1663208 | 1663208 | 1011095 | 1011095 | 1011277 | 2436057 | 2436057 | 2436057 |
| GC (%) |  | 47.46 | 47.46 | 47.46 | 47.46 | 47.61 | 47.63 | 47.56 | 48.16 | 48.15 | 48.15 |
| N50 |  | 20933 | 23627 | 23801 | 23801 | 11714 | 11742 | 11754 | 12296 | 12363 | 12366 |
| N75 |  | 9319 | 10264 | 10294 | 10293 | 4596 | 4607 | 4570 | 4885 | 4932 | 4936 |
| L50 |  | 5247 | 4723 | 4700 | 4700 | 6959 | 6947 | 6880 | 7301 | 7239 | 7240 |
| L75 |  | 13674 | 12297 | 12249 | 12248 | 19740 | 19723 | 19606 | 21576 | 21389 | 21390 |
| # N's per 100 kbp |  | 0.00 | 80.46 | 80.53 | 22.06 | 3361.65 | 3358.04 | 24.44 | 80.36 | 80 | 5.44 |
|  |  |  |  |  |  |  |  |  |  |  |  |
| All statistics are based on sequences of size >= 1000 bp | | | | |  |  |  |  |  |  |  |
