# Supplementary material for: The first draft genome of the aquatic model plant Lemna minor opens the route for future stress physiology research and biotechnological applications
Source: Biotechnol Biofuels. 2015 Nov 25;8:188. doi: 10.1186/s13068-015-0381-1 (PMC4659200; doi:10.1186/s13068-015-0381-1)
Supplement: Supplementary file 3 — 10.1186/s13068-015-0381-1 Number of CEGMA hits for different genome assemblies. [file 13068_2015_381_MOESM3_ESM.docx]

**Supplementary Table S3:** number of CEGMA hits for different genome assemblies

|  | number of proteins with  complete coverage (> 70%) | percentage (%) | number of proteins with  partial coverage | | | percentage (%) |  |
| --- | --- | --- | --- | --- | --- | --- | --- |
| CLC assembly | 203 | 81,85 | |  | 230 | 92,74 | |
| SOAP assembly | 197 | 79,44 | |  | 231 | 93,15 | |
| Masurca assembly | 215 | 86,69 | |  | 233 | 93,95 | |
|  |  |  | |  |  |  | |
| masurca for contigs > 2kbp | 213 | 85,08 | |  | 230 | 92,74 | |
