# Supplementary material for: The first draft genome of the aquatic model plant Lemna minor opens the route for future stress physiology research and biotechnological applications
Source: Biotechnol Biofuels. 2015 Nov 25;8:188. doi: 10.1186/s13068-015-0381-1 (PMC4659200; doi:10.1186/s13068-015-0381-1)
Supplement: Supplementary file 4 — 10.1186/s13068-015-0381-1 Statistics of transcriptome and whole genome assembly of L. minor.. [file 13068_2015_381_MOESM4_ESM.docx]

**Supplementary Table S4:** statistics of transcriptome and whole genome assembly of *L. minor*

| \| Description \|  \| percentage (%) \| \| --- \| --- \| --- \| \| Total number of transcripts assembled* \| 22382 \|  \| \| Number of transcripts with 100% coverage in whole genome \| 17302 \| 77,30 \| \| Number of transcripts with 95% coverage in whole genome \| 21739 \| 97,13 \| \| Number of transcripts with 90% coverage in whole genome \| 22041 \| 98,48 \| \| Number of transcripts with 85% coverage in whole genome \| 22222 \| 99,29 \| \| Number of transcripts with 80% coverage in whole genome \| 22231 \| 99,33 \| \| Number of transcripts with 70% coverage in whole genome \| 22243 \| 99,38 \| |  |  |
| --- | --- | --- | --- | --- | --- | --- | --- | --- | --- | --- | --- | --- | --- | --- | --- | --- | --- | --- | --- | --- | --- | --- | --- | --- | --- | --- |
|  |  |  |
|  |  |  |
|  |  |  |
|  |  |  |
|  |  |  |
|  |  |  |
|  |  |  |
