# Supplementary figures and images for: The first draft genome of the aquatic model plant Lemna minor opens the route for future stress physiology research and biotechnological applications
Source: Biotechnol Biofuels. 2015 Nov 25;8:188. doi: 10.1186/s13068-015-0381-1 (PMC4659200; doi:10.1186/s13068-015-0381-1)

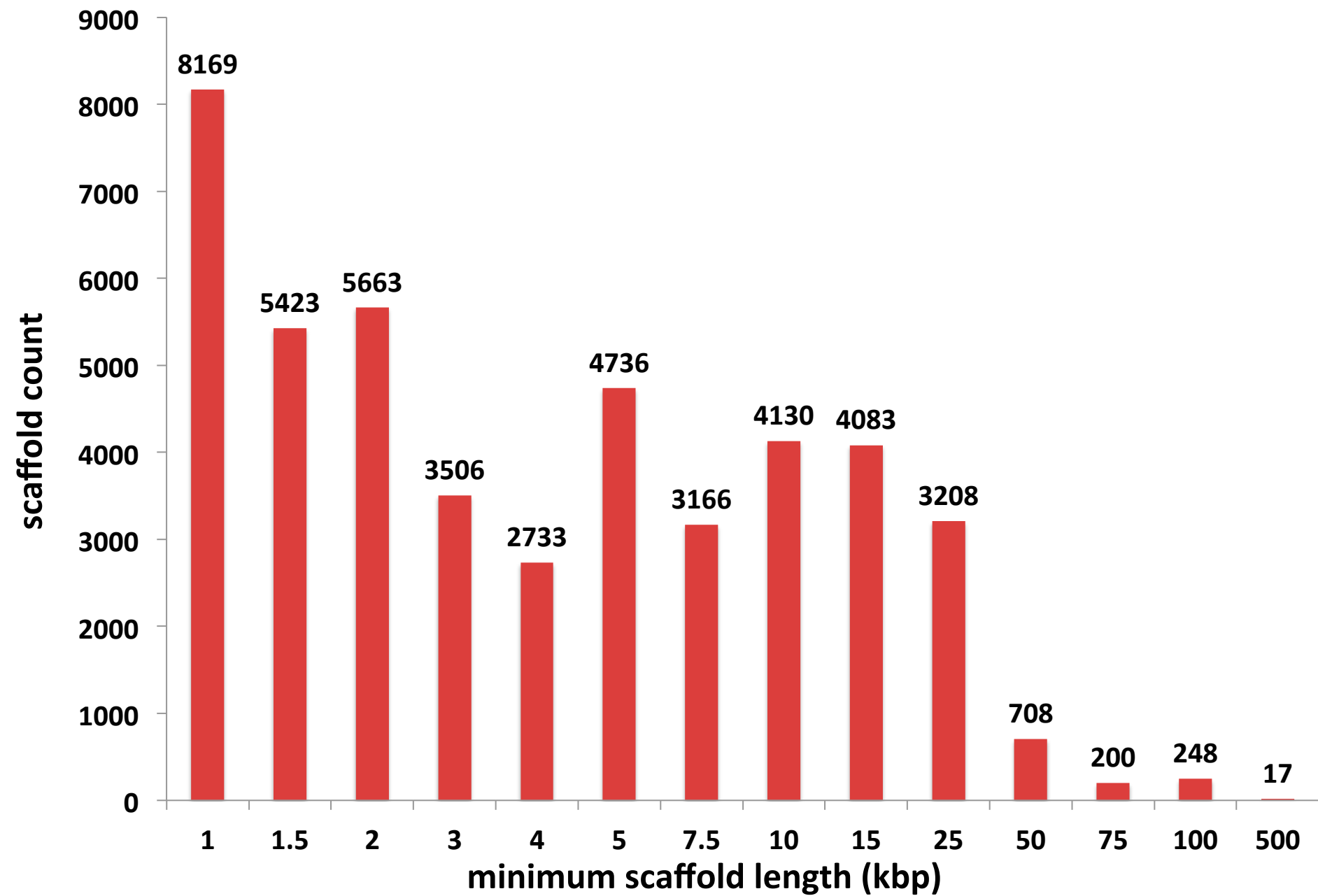

Supplement: Supplementary file 5 — 10.1186/s13068-015-0381-1 Assembled scaffold length distribution of L. minor genome. [file 13068_2015_381_MOESM5_ESM.pdf]

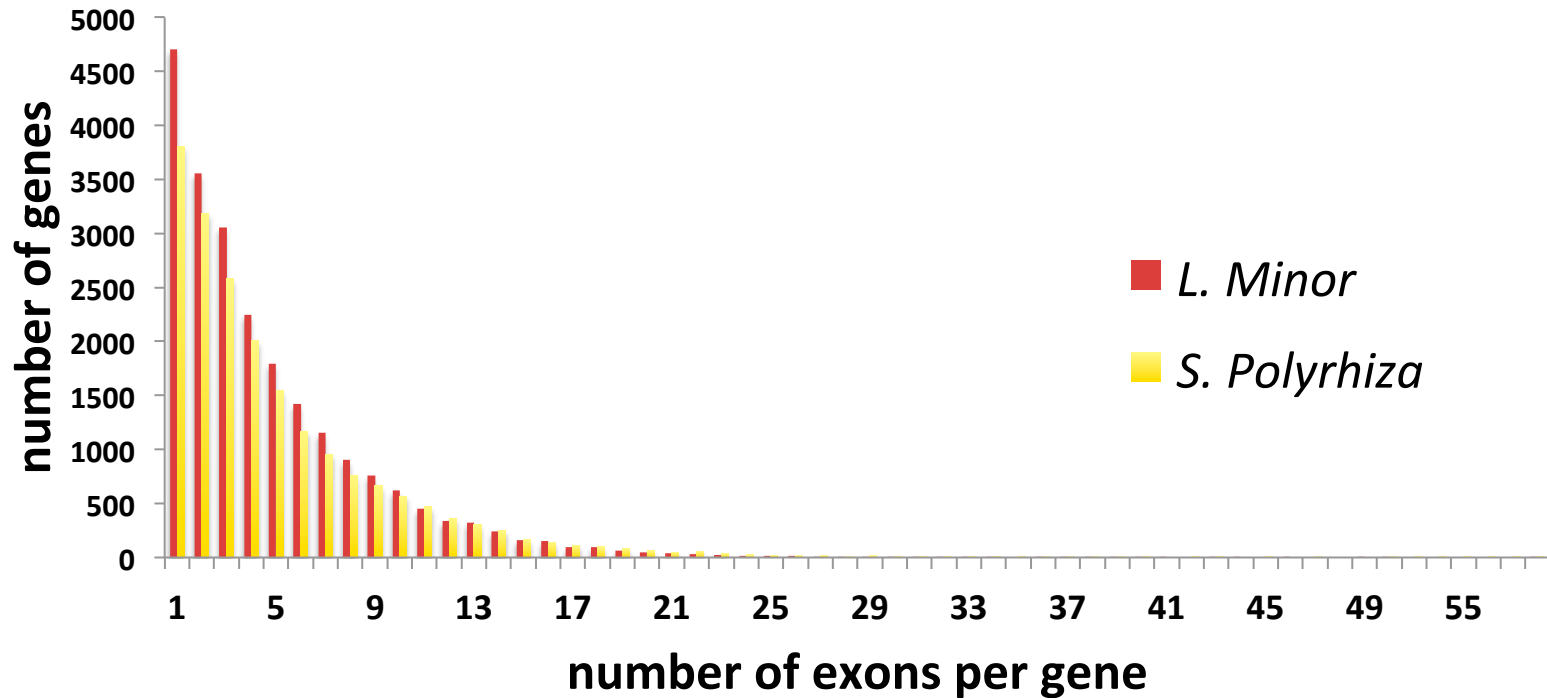

Supplement: Supplementary file 9 — 10.1186/s13068-015-0381-1 Distribution of the number of exons per gene. [file 13068_2015_381_MOESM9_ESM.pdf]

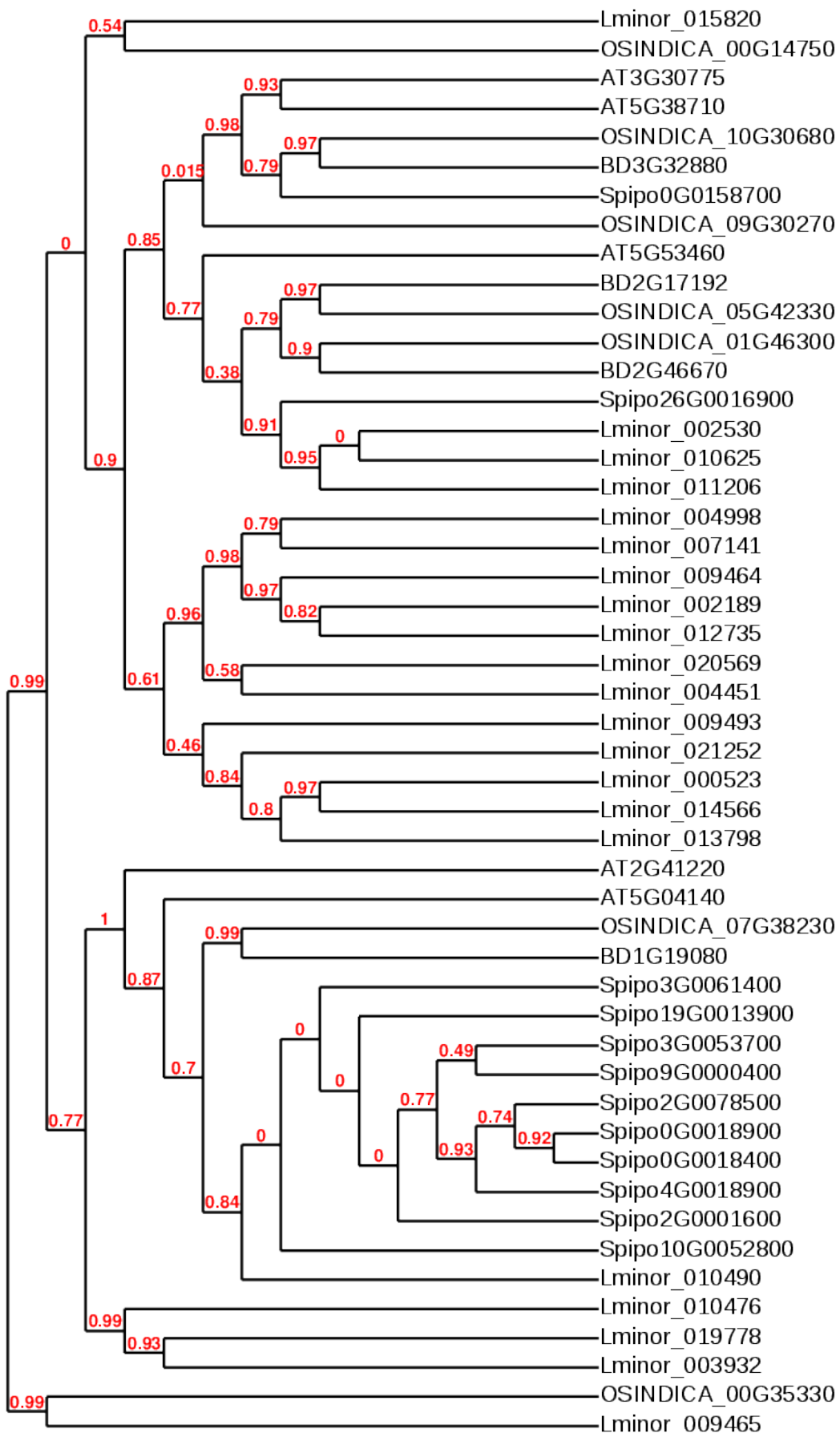

Supplement: Supplementary file 15 — 10.1186/s13068-015-0381-1 Cladogram of glutamate synthase isoforms in L. minor (Lminor), S. polyrhiza (Spipo), A. thaliana (AT), O. indica (OSINDICA) and B. distachyon (BD). The phylogentic relationships of glutamate synthase from different species have been calculated under default parameters using phylogeny.fr. [file 13068_2015_381_MOESM15_ESM.pdf]

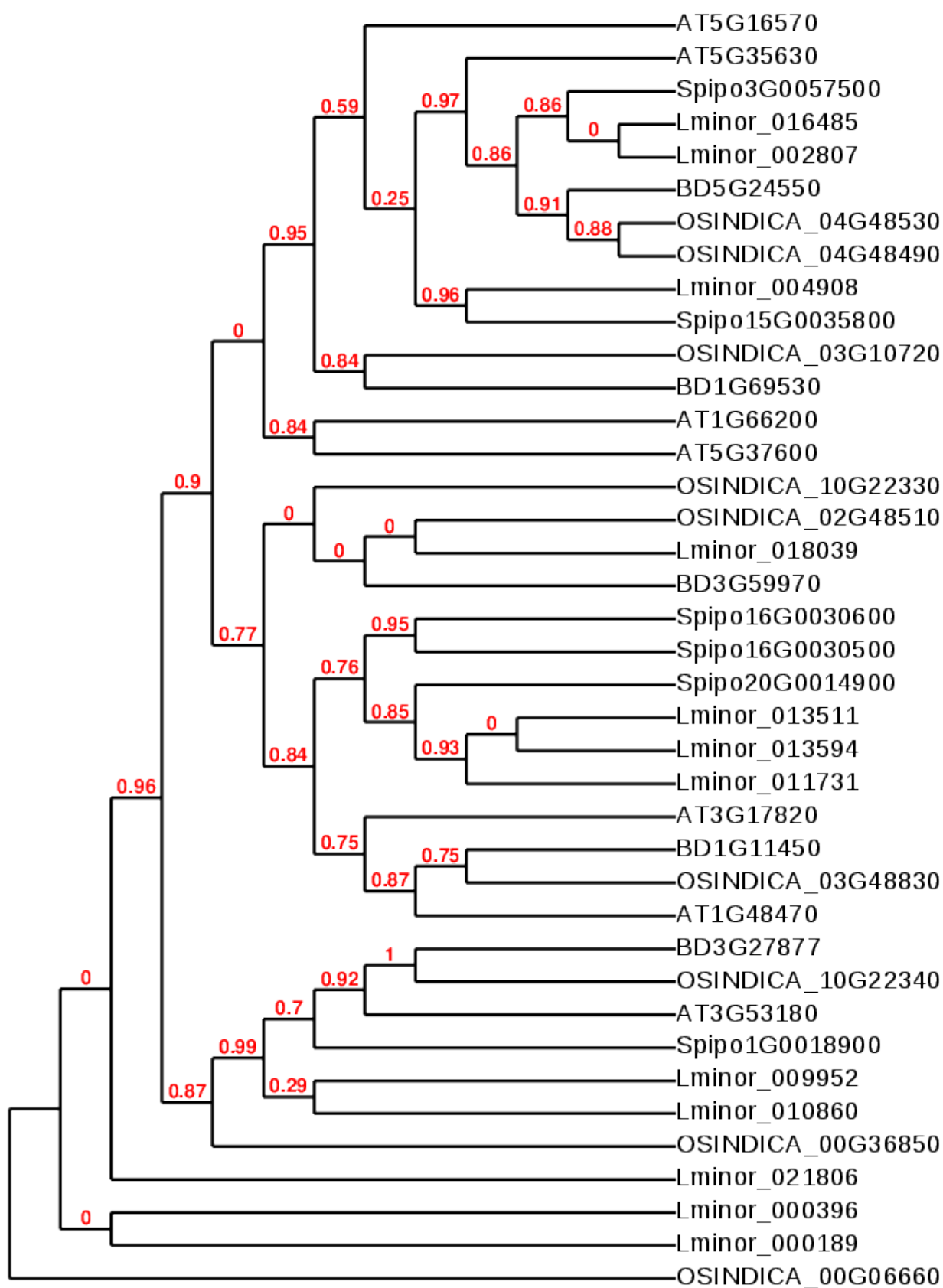

Supplement: Supplementary file 16 — 10.1186/s13068-015-0381-1 Cladogram of glutamine synthetase isoform in L. minor (Lminor), S. polyrhiza (Spipo), A. thaliana (AT), O. indica (OSINDICA) and B. distachyon (BD). The phylogentic relationships of glutamine synthetase from different species have been calculated under default parameters using phylogeny.fr. [file 13068_2015_381_MOESM16_ESM.pdf]
