# Supplementary material for: The first draft genome of the aquatic model plant Lemna minor opens the route for future stress physiology research and biotechnological applications
Source: Biotechnol Biofuels. 2015 Nov 25;8:188. doi: 10.1186/s13068-015-0381-1 (PMC4659200; doi:10.1186/s13068-015-0381-1)
Supplement: Supplementary file 6 — 10.1186/s13068-015-0381-1 Variant calling of L. minor (strain 5500) chloroplast genome vs. L. minor GenBank reference genome NC_01019. [file 13068_2015_381_MOESM6_ESM.docx]

**Supplementary Table S5:** variant calling of *L. minor* chloroplast genome vs. *L. minor* GenBank reference genome NC_01019

| Genbank L. Minor chloroplast reference | POS | ID | REF | ALT | QUAL | FILTER | INFO | FORMAT | Lemna clone 5500 |
| --- | --- | --- | --- | --- | --- | --- | --- | --- | --- |
| NC_010109 | 84 | . | A | AT | . | . | . | GT:CLCAD2:DP | 1/1:0,40:78 |
| NC_010109 | 92 | . | T | TA | . | . | . | GT:CLCAD2:DP | 1/1:0,58:87 |
| NC_010109 | 93 | . | T | TA | . | . | . | GT:CLCAD2:DP | 1/1:0,58:87 |
| NC_010109 | 97 | . | A | T | . | . | . | GT:CLCAD2:DP | 1/1:0,58:86 |
| NC_010109 | 98 | . | AT | A | . | . | . | GT:CLCAD2:DP | 1/1:0,58:84 |
| NC_010109 | 101 | . | TA | T | . | . | . | GT:CLCAD2:DP | 1/1:0,58:83 |
| NC_010109 | 110 | . | A | T | . | . | . | GT:CLCAD2:DP | 1/1:0,86:111 |
| NC_010109 | 113 | . | T | A | . | . | . | GT:CLCAD2:DP | 1/1:0,110:114 |
| NC_010109 | 1387 | . | G | T | . | . | . | GT:CLCAD2:DP | 1/1:0,15386:15469 |
| NC_010109 | 2159 | . | C | T | . | . | . | GT:CLCAD2:DP | 1/1:0,8895:8912 |
| NC_010109 | 6680 | . | C | T | . | . | . | GT:CLCAD2:DP | 1/1:0,6507:6586 |
| NC_010109 | 7686 | . | C | T | . | . | . | GT:CLCAD2:DP | 1/1:0,10317:10323 |
| NC_010109 | 9631 | . | T | C | . | . | . | GT:CLCAD2:DP | 1/1:0,2901:2924 |
| NC_010109 | 9745 | . | T | A | . | . | . | GT:CLCAD2:DP | 1/1:0,1185:1188 |
| NC_010109 | 10033 | . | AGTAGAATTT  TTTATGTACA  ACTTAAATAG  CTTCTTCAAG | A | . | . | . | GT:CLCAD2:DP | 1/1:0,454:500 |
| NC_010109 | 10738 | . | C | CT | . | . | . | GT:CLCAD2:DP | 1/1:0,9202:9953 |
| NC_010109 | 10883 | . | C | T | . | . | . | GT:CLCAD2:DP | 1/1:0,10908:10980 |
| NC_010109 | 13700 | . | T | A | . | . | . | GT:CLCAD2:DP | 1/1:0,4554:4569 |
| NC_010109 | 15481 | . | T | A | . | . | . | GT:CLCAD2:DP | 1/1:0,8240:8262 |
| NC_010109 | 29122 | . | G | T | . | . | . | GT:CLCAD2:DP | 1/1:0,5162:5263 |
| NC_010109 | 29967 | . | C | CA | . | . | . | GT:CLCAD2:DP | 1/1:0,5365:6740 |
| NC_010109 | 32676 | . | G | GTACTAAA | . | . | . | GT:CLCAD2:DP | 0/1:2578,5349:7929 |
| NC_010109 | 32989 | . | A | T | . | . | . | GT:CLCAD2:DP | 1/1:0,2325:2345 |
| NC_010109 | 35529 | . | G | T | . | . | . | GT:CLCAD2:DP | 1/1:0,7546:7714 |
| NC_010109 | 39605 | . | C | CCGTGAG | . | . | . | GT:CLCAD2:DP | 0/1:3017,6612:9643 |
| NC_010109 | 39955 | . | AG | A | . | . | . | GT:CLCAD2:DP | 1/1:0,10763:11144 |
| NC_010109 | 47911 | . | TCAATGAAG | T | . | . | . | GT:CLCAD2:DP | 1/1:0,5225:5265 |
| NC_010109 | 48765 | . | TGGTATAAAA | T | . | . | . | GT:CLCAD2:DP | 1/1:0,5931:5968 |
| NC_010109 | 51990 | . | GA | G | . | . | . | GT:CLCAD2:DP | 1/1:0,2500:2965 |
| NC_010109 | 55607 | . | T | A | . | . | . | GT:CLCAD2:DP | 1/1:0,664:818 |
| NC_010109 | 61938 | . | G | T | . | . | . | GT:CLCAD2:DP | 1/1:0,10056:10080 |
| NC_010109 | 67902 | . | CT | C | . | . | . | GT:CLCAD2:DP | 1/1:0,9909:10722 |
| NC_010109 | 70708 | . | AC | A | . | . | . | GT:CLCAD2:DP | 1/1:0,8078:8501 |
| NC_010109 | 73661 | . | A | C | . | . | . | GT:CLCAD2:DP | 1/1:0,10188:10949 |
| NC_010109 | 83017 | . | TC | T | . | . | . | GT:CLCAD2:DP | 1/1:0,10160:10532 |
| NC_010109 | 123876 | . | T | G | . | . | . | GT:CLCAD2:DP | 1/1:0,3543:3550 |
| NC_010109 | 124095 | . | G | T | . | . | . | GT:CLCAD2:DP | 1/1:0,6657:6713 |
| NC_010109 | 127498 | . | C | T | . | . | . | GT:CLCAD2:DP | 1/1:0,10211:10301 |
| NC_010109 | 129754 | . | C | T | . | . | . | GT:CLCAD2:DP | 1/1:0,5374:5381 |
| NC_010109 | 130660 | . | G | GT | . | . | . | GT:CLCAD2:DP | 1/1:0,1159:1199 |
| NC_010109 | 149320 | . | A | T | . | . | . | GT:CLCAD2:DP | 1/1:0,24:26 |
| NC_010109 | 149321 | . | C | CA | . | . | . | GT:CLCAD2:DP | 1/1:0,24:26 |
| NC_010109 | 149323 | . | TC | T | . | . | . | GT:CLCAD2:DP | 1/1:0,24:26 |
| NC_010109 | 149331 | . | GC | AT | . | . | . | GT:CLCAD2:DP | 1/1:0,24:26 |
| NC_010109 | 149339 | . | CCG | GGA | . | . | . | GT:CLCAD2:DP | 1/1:0,24:26 |
| NC_010109 | 149344 | . | A | G | . | . | . | GT:CLCAD2:DP | 1/1:0,24:26 |
| NC_010109 | 149360 | . | CGG | TCC | . | . | . | GT:CLCAD2:DP | 1/1:0,24:26 |
| NC_010109 | 149365 | . | A | G | . | . | . | GT:CLCAD2:DP | 1/1:0,24:26 |
