# Supplementary material for: The first draft genome of the aquatic model plant Lemna minor opens the route for future stress physiology research and biotechnological applications
Source: Biotechnol Biofuels. 2015 Nov 25;8:188. doi: 10.1186/s13068-015-0381-1 (PMC4659200; doi:10.1186/s13068-015-0381-1)
Supplement: Supplementary file 7 — 10.1186/s13068-015-0381-1 Summary of the masked and unmasked genome assembly. [file 13068_2015_381_MOESM7_ESM.docx]

**Supplementary Table S6:** summary of the masked and unmasked genome assembly

|  | contigs |  |  |  |  |  | contigs > 2kbp | |  |  |  |
| --- | --- | --- | --- | --- | --- | --- | --- | --- | --- | --- | --- |
|  | **unmasked** |  |  | **masked** |  |  | **unmasked** |  |  | **masked** |  |
|  |  |  |  |  |  |  |  |  |  |  |  |
| A | 124033571 | 26% |  | 44149090 | 9% |  | 119025647 | 26% |  | 42785196 | 9% |
| C | 111998225 | 24% |  | 44688687 | 9% |  | 107214034 | 24% |  | 43258164 | 10% |
| G | 112041572 | 24% |  | 44637501 | 9% |  | 107257785 | 24% |  | 43213910 | 10% |
| N | 104150 | 0,02% |  | 294552973 | 62% |  | 100939 | 0,02% |  | 280542917 | 62% |
| T | 123951185 | 26% |  | 44100452 | 9% |  | 118929953 | 26% |  | 42728171 | 9% |
| aA | 124033571 | 26% |  | 44149090 | 9% |  | 119025647 | 26% |  | 42785196 | 9% |
| aAtT | 247984756 | 53% |  | 88249542 | 19% |  | 237955600 | 53% |  | 85513367 | 19% |
| aAtTgGcC | 472024553 | 100% |  | 177575730 | 38% |  | 452427419 | 100% |  | 171985441 | 38% |
| cC | 111998225 | 24% |  | 44688687 | 9% |  | 107214034 | 24% |  | 43258164 | 10% |
| gG | 112041572 | 24% |  | 44637501 | 9% |  | 107257785 | 24% |  | 43213910 | 10% |
| gGcC | 224039797 | 47% |  | 89326188 | 19% |  | 214471819 | 47% |  | 86472074 | 19% |
| nN | 104150 | 0,02% |  | 294552973 | 62% |  | 100939 | 0,02% |  | 280542917 | 62% |
| tT | 123951185 | 26% |  | 44100452 | 9% |  | 118929953 | 26% |  | 42728171 | 9% |
|  |  |  |  |  |  |  |  |  |  |  |  |
| Total nts | 472128703 |  |  | 472128703 |  |  | 452528358 |  |  | 452528358 |  |
