# Supplementary material for: The first draft genome of the aquatic model plant Lemna minor opens the route for future stress physiology research and biotechnological applications
Source: Biotechnol Biofuels. 2015 Nov 25;8:188. doi: 10.1186/s13068-015-0381-1 (PMC4659200; doi:10.1186/s13068-015-0381-1)
Supplement: Supplementary file 8 — 10.1186/s13068-015-0381-1 Illumina libraries statistics for transcriptome assembly. [file 13068_2015_381_MOESM8_ESM.docx]

**Supplementary Table S7:** Illumina libraries statistics for transcriptome assembly

| **stress condition 1** | **sample** | **RIN** | **ng/ul** |  | **sequenced** | **quality cutt off = 20 (90%)** | **trimmed reads** | **clipped <= 20 bp** | **remove artifacts** | **% non-discarded** |
| --- | --- | --- | --- | --- | --- | --- | --- | --- | --- | --- |
| control 1 | 1 | 9 | 178 |  | 17267134 | 16151348 | 719973 | 14607930 | 14607875 | 84,5993029 |
| control 2 | 2 | 9 | 166 |  | 13408247 | 12472226 | 542778 | 11250561 | 11250466 | 83,9070611 |
| control 3 | 3 | 9,1 | 308 |  | 11982123 | 11191765 | 495787 | 10118174 | 10118022 | 84,4426484 |
| conditie A 1 | 5 | 8,9 | 191 |  | 13278461 | 12342818 | 543256 | 11145606 | 11145425 | 83,9361203 |
| conditie A 2 | 6 | 8,9 | 291 |  | 11001022 | 10279331 | 462846 | 9309775 | 9309517 | 84,6241104 |
| conditie A 3 | 7 | 8,9 | 246 |  | 11040225 | 10347787 | 472907 | 9398290 | 9398030 | 85,1253484 |
| conditie B 1 | 9 | 8,9 | 282 |  | 16797239 | 15661129 | 691260 | 14162055 | 14161980 | 84,3113562 |
| conditie B 2 | 10 | 9 | 287 |  | 13778269 | 12813712 | 557328 | 11564775 | 11564655 | 83,9340196 |
| conditie B 3 | 11 | 9 | 344 |  | 13278461 | 12342818 | 543256 | 11145606 | 11145425 | 83,9361203 |
| conditie C 1 | 13 | 8,6 | 397 |  | 13601246 | 12585124 | 537807 | 11338566 | 11338513 | 83,3637815 |
| conditie C 2 | 14 | 8,9 | 473 |  | 12154299 | 11257516 | 478051 | 10138888 | 10138852 | 83,4178261 |
| conditie C 3 | 15 | 10 | 197 |  | 13545884 | 12573884 | 528156 | 11333859 | 11333815 | 83,669807 |
| conditie D 1 | 17 | 8,8 | 224 |  | 11231430 | 10451073 | 461867 | 9435003 | 9434973 | 84,0050911 |
| conditie D 2 | 18 | 8,5 | 454 |  | 8754381 | 8173982 | 363439 | 7393768 | 7393696 | 84,4570964 |
| conditie D 3 | 19 | 8,5 | 488 |  | 14213657 | 13252899 | 590825 | 11979594 | 11979503 | 84,2816384 |
|  |  |  |  |  |  |  |  |  |  |  |
| **stress condition 2** |  |  |  |  | **sequenced** | **quality cutt off = 20 (90%)** | **trimmed reads** | **clipped <= 20 bp** | **remove artifacts** | **% non-discarded** |
| control 2 | 22 | 8,4 | 68 |  | 11828492 | 11066706 | 502378 | 10040148 | 10039982 | 84,8796448 |
| control 3 | 23 | 8,6 | 209 |  | 13641022 | 12763439 | 581725 | 11584198 | 11584107 | 84,9211078 |
| control 4 | 24 | 7,5 | 167 |  | 10804691 | 10122242 | 467693 | 9196054 | 9195952 | 85,1107357 |
| conditie A 1 | 25 | 8,4 | 170 |  | 12468562 | 11672243 | 532956 | 10581755 | 10581719 | 84,8671964 |
| conditie A 2 | 26 | 9 | 206 |  | 12563962 | 11740342 | 529118 | 10627488 | 10627379 | 84,5862078 |
| conditie A 3 | 27 | 8,5 | 193 |  | 13793378 | 12815381 | 555471 | 11554237 | 11554116 | 83,7656736 |
| conditie B 1 | 29 | 7,8 | 49 |  | 16930865 | 15749642 | 676521 | 14187289 | 14187195 | 83,7948622 |
| conditie B 2 | 30 | 7,5 | 46 |  | 16665785 | 15502862 | 681655 | 13986110 | 13986030 | 83,9206194 |
| conditie B 3 | 31 | 7,7 | 41 |  | 13061046 | 12103296 | 528230 | 10884735 | 10884640 | 83,3366638 |
| conditie C 1 | 33 | 7,4 | 33 |  | 12391441 | 11983753 | 600128 | 10926290 | 10925656 | 88,1709883 |
| conditie C 2 | 34 | 7,5 | 57 |  | 21492737 | 20693028 | 954318 | 18734348 | 18733839 | 87,16358 |
| conditie C 3 | 35 | 7,2 | 55 |  | 11851628 | 11449460 | 544291 | 10417963 | 10417674 | 87,9007846 |
| conditie D 1 | 37 | 7,2 | 35 |  | 11937796 | 11514244 | 546390 | 10460901 | 10460323 | 87,6235697 |
| conditie D 2 | 38 | 6,7 | 25 |  | 11562162 | 11172674 | 533723 | 10180559 | 10180269 | 88,0481436 |
| conditie D 3 | 39 | 7,5 | 19 |  | 13242486 | 12806824 | 629966 | 11695933 | 11695577 | 88,3185906 |
|  |  |  |  |  |  |  |  |  |  |  |
|  |  |  |  |  |  |  |  |  |  |  |
| **stress condition 3** |  |  |  |  | **sequenced** | **quality cutt off = 20 (90%)** | **trimmed reads** | **clipped <= 20 bp** | **remove artifacts** | **% non-discarded** |
| control 1 | 42 | 8,4 | 116 |  | 17917421 | 17305357 | 831059 | 15752935 | 15752685 | 87,9182612 |
| control 2 | 43 | 8,4 | 119 |  | 13484232 | 12997881 | 606669 | 11794619 | 11794431 | 87,4683186 |
| control 3 | 44 | 8,4 | 110 |  | 21958099 | 21119836 | 953308 | 19110678 | 19110446 | 87,0314229 |
| conditie A 1 | 45 | 8,2 | 142 |  | 14430212 | 13834669 | 607476 | 12500921 | 12500834 | 86,6295935 |
| conditie A 2 | 46 | 8,2 | 110 |  | 14810383 | 14204855 | 625064 | 12833079 | 12832932 | 86,6482116 |
| conditie A 3 | 48 | 8,4 | 253 |  | 21594110 | 20720836 | 901886 | 18690236 | 18690114 | 86,551907 |
| conditie B 1 | 49 | 8,5 | 330 |  | 13331720 | 12794811 | 560563 | 11555877 | 11555823 | 86,6791607 |
| conditie B 2 | 51 | 7,6 | 372 |  | 18162604 | 17495877 | 816572 | 15872145 | 15871930 | 87,3879649 |
| conditie B 3 | 52 | 8,5 | 349 |  | 16966211 | 16359564 | 771375 | 14841965 | 14841665 | 87,4777816 |
| conditie C 1 | 54 | 8,4 | 257 |  | 14695379 | 14142967 | 638681 | 12799556 | 12799363 | 87,0978761 |
| conditie C 2 | 56 | 8,1 | 206 |  | 16583344 | 15988583 | 734866 | 14498533 | 14498324 | 87,4270232 |
| conditie C 3 | 70 | 7,8 | 530 |  | 12993653 | 12462878 | 538981 | 11233575 | 11233516 | 86,453871 |
| conditie D 1 | 57 | 8,4 | 214 |  | 17091086 | 16477538 | 761838 | 14951147 | 14951004 | 87,4783732 |
| conditie D 2 | 59 | 8,1 | 336 |  | 15469666 | 14866479 | 668057 | 13438522 | 13438441 | 86,869626 |
| conditie D 3 | 60 | 8,2 | 300 |  | 16166580 | 15564942 | 714279 | 14078238 | 14078102 | 87,0815101 |
| conditie E 1 | 61 | 7,9 | 121 |  | 14384176 | 13820280 | 623419 | 12492139 | 12491766 | 86,8438067 |
| conditie E 2 | 63 | 7,1 | 292 |  | 17001963 | 16340981 | 725577 | 14731345 | 14731252 | 86,6444186 |
| conditie E 3 | 64 | 8,2 | 265 |  | 13598911 | 13062918 | 563984 | 11778644 | 11778569 | 86,6140605 |
