# Supplementary material for: The first draft genome of the aquatic model plant Lemna minor opens the route for future stress physiology research and biotechnological applications
Source: Biotechnol Biofuels. 2015 Nov 25;8:188. doi: 10.1186/s13068-015-0381-1 (PMC4659200; doi:10.1186/s13068-015-0381-1)
Supplement: Supplementary file 10 — 10.1186/s13068-015-0381-1 BLAST results of L. minor GenBank genes vs L. minor 5500. [file 13068_2015_381_MOESM10_ESM.docx]

**Supplementary Table S8:** BLAST (NCBI) results of *. minor* GenBank genes vs *L. minor* 5500

| **Lemna prot GenBank** | **Lemna minor 5500** | **E-value** |
| --- | --- | --- |
| gi\|584594525\|gb\|AHJ10960.1\| | Lminor_004908 | 0,0 |
| gi\|584594523\|gb\|AHJ10959.1\| | Lminor_004908 | 0,0 |
| gi\|484265031\|emb\|CCW44110.1\| | Lminor_004348 | 0,0 |
| gi\|484265029\|emb\|CCW44109.1\| | Lminor_004348 | 0,0 |
| gi\|484265027\|emb\|CCW44108.1\| | Lminor_004348 | 0,0 |
| gi\|484265025\|emb\|CCW44107.1\| | Lminor_004348 | 0,0 |
| gi\|484265023\|emb\|CCW44106.1\| | Lminor_002807 | 0,0 |
| gi\|484265021\|emb\|CCW44105.1\| | Lminor_002807 | 0,0 |
| gi\|484265019\|emb\|CCW44104.1\| | Lminor_002807 | 0,0 |
| gi\|484265017\|emb\|CCW44103.1\| | Lminor_002807 | 0,0 |
| gi\|484265014\|emb\|CCW44102.1\| | Lminor_004908 | 0,0 |
| gi\|484265012\|emb\|CCW44101.1\| | Lminor_004908 | 0,0 |
| gi\|484265010\|emb\|CCW44100.1\| | Lminor_004908 | 0,0 |
| gi\|484265008\|emb\|CCW44099.1\| | Lminor_004908 | 0,0 |
| gi\|484265006\|emb\|CCW44098.1\| | Lminor_003252 | 0,0 |
| gi\|484265004\|emb\|CCW44097.1\| | Lminor_003252 | 0,0 |
| gi\|484265002\|emb\|CCW44096.1\| | Lminor_003252 | 0,0 |
| gi\|484265000\|emb\|CCW44095.1\| | Lminor_003252 | 0,0 |
| gi\|350286133\|gb\|AEQ28336.1\| | Lminor_003252 | 0,0 |
| gi\|300654362\|emb\|CBV02242.1\| | Lminor_016295 | 0,0 |
| gi\|259456338\|emb\|CBF86616.1\| | Lminor_016295 | 0,0 |
| gi\|218614160\|emb\|CAV32821.1\| | Lminor_012770 | 0,0 |
| gi\|218614145\|emb\|CAV32819.1\| | Lminor_012770 | 0,0 |
| gi\|218614143\|emb\|CAV32817.1\| | Lminor_012770 | 0,0 |
| gi\|218614141\|emb\|CAV32814.1\| | Lminor_015244 | 0,0 |
| gi\|218614139\|emb\|CAV32813.1\| | Lminor_015244 | 0,0 |
| gi\|218465403\|emb\|CAV30454.1\| | Lminor_012770 | 0,0 |
| gi\|218465388\|emb\|CAV30453.1\| | Lminor_012770 | 0,0 |
| gi\|218465386\|emb\|CAV30452.1\| | Lminor_012770 | 0,0 |
| gi\|218465384\|emb\|CAV30451.1\| | Lminor_015244 | 0,0 |
| gi\|218465382\|emb\|CAV30450.1\| | Lminor_015244 | 0,0 |
| gi\|110735396\|gb\|ABG89268.1\| | Lminor_015244 | 0,0 |
| gi\|110735394\|gb\|ABG89267.1\| | Lminor_012770 | 0,0 |
| gi\|15209622\|emb\|CAC51139.1\| | Lminor_016295 | 0,0 |
| gi\|6706278\|emb\|CAB65911.1\| | Lminor_016295 | 0,0 |
| gi\|50660440\|gb\|AAT80908.1\| | Lminor_004152 | 2,00E-144 |
| gi\|254914197\|gb\|ACT83792.1\| | Lminor_005112 | 1,00E-107 |
| gi\|240064589\|gb\|ACS44642.1\| | Lminor_020356 | 8,00E-102 |
| gi\|254914195\|gb\|ACT83791.1\| | Lminor_011285 | 3,00E-101 |
| gi\|144953503\|gb\|ABP04114.1\| | Lminor_018704 | 3,00E-95 |
| gi\|187438937\|gb\|ACD10928.1\| | Lminor_009106 | 4,00E-87 |
| gi\|50660432\|gb\|AAT80904.1\| | Lminor_000548 | 1,00E-81 |
| gi\|187438933\|gb\|ACD10926.1\| | Lminor_007663 | 4,00E-81 |
| gi\|50660436\|gb\|AAT80906.1\| | Lminor_001230 | 1,00E-80 |
| gi\|145203147\|gb\|ABP35940.1\| | Lminor_018704 | 3,00E-77 |
| gi\|110735398\|gb\|ABG89269.1\| | Lminor_012770 | 1,00E-70 |
| gi\|254914199\|gb\|ACT83793.1\| | Lminor_010610 | 2,00E-63 |
| gi\|148615526\|gb\|ABQ96601.1\| | Lminor_016065 | 6,00E-59 |
| gi\|254914201\|gb\|ACT83794.1\| | Lminor_017198 | 3,00E-57 |
| gi\|50660438\|gb\|AAT80907.1\| | Lminor_010805 | 8,00E-49 |
| gi\|807059055\|gb\|AKC42390.1\| | Lminor_002877 | 2,00E-44 |
| gi\|254914191\|gb\|ACT83789.1\| | Lminor_011360 | 2,00E-44 |
| gi\|187438935\|gb\|ACD10927.1\| | Lminor_009150 | 5,00E-44 |
| gi\|209417451\|emb\|CAP19732.1\| | Lminor_011126 | 1,00E-42 |
| gi\|187470377\|gb\|ACD11149.1\| | Lminor_002736 | 1,00E-41 |
| gi\|254914193\|gb\|ACT83790.1\| | Lminor_012775 | 7,00E-39 |
| gi\|50660434\|gb\|AAT80905.1\| | Lminor_005331 | 3,00E-33 |
| gi\|50660442\|gb\|AAT80909.1\| | Lminor_018463 | 6,00E-28 |
| gi\|50660430\|gb\|AAT80903.1\| | Lminor_010537 | 9,00E-16 |
| gi\|240064638\|gb\|ACS44644.1\| | Lminor_010705 | 8,00E-14 |
| gi\|209417651\|emb\|CAP58306.1\| | Lminor_015182 | 0,048 |
