# Supplementary material for: The first draft genome of the aquatic model plant Lemna minor opens the route for future stress physiology research and biotechnological applications
Source: Biotechnol Biofuels. 2015 Nov 25;8:188. doi: 10.1186/s13068-015-0381-1 (PMC4659200; doi:10.1186/s13068-015-0381-1)
Supplement: Supplementary file 11 — 10.1186/s13068-015-0381-1 Overview of OrthoMCL gene clusters for L. minor with S. polyrhiza, Z. mays and O. sativa. [file 13068_2015_381_MOESM11_ESM.docx]

**Supplementary Table S9:** overview of OrthoMCL gene clusters for *L. minor* with *S. polyrhiza, Z. mays* and *O. sativa*

|  | ***S. polyrhiza*** | ***L. minor*** | ***Z. mays*** | ***O. sativa*** |
| --- | --- | --- | --- | --- |
| Total genes | 19.623 | 22.382 | 39.305 | 48.788 |
| Orphan genes | 3.918 | 3.546 | 9.850 | 15.826 |
| % orphan genes | 0,20 | 0,16 | 0,25 | 0,32 |
|  |  |  |  |  |
| Overal clusters |  |  |  |  |
| Clustered genes | 15.705 | 18.836 | 29.455 | 32.962 |
| Clustered clusters | 11.629 | 11.663 | 15.764 | 16.399 |
| Average genes/clusters | 1,35 | 1,62 | 1,87 | 2,01 |
|  |  |  |  |  |
| Species-specific |  |  |  |  |
| Clustered genes | 1.511 | 2.897 | 5.450 | 9.267 |
| Clustered clusters | 352 | 795 | 1.741 | 2.298 |
| Average genes/clusters | 4,29 | 3,64 | 3,13 | 4,03 |
|  |  |  |  |  |
| Duckweed-specific |  |  |  |  |
| Clustered genes | 1.546 | 1.821 |  |  |
| Clustered clusters | 1.356 | 1.356 |  |  |
| Average genes/clusters | 1,14 | 1,34 |  |  |
|  |  |  |  |  |
| All species-presence |  |  |  |  |
| Clustered genes | 10.627 | 12.128 | 15.090 | 13.132 |
| Clustered clusters | 8.202 | 8.202 | 8.202 | 8.202 |
| Average genes/clusters | 1,30 | 1,48 | 1,84 | 1,60 |
