# Supplementary material for: The first draft genome of the aquatic model plant Lemna minor opens the route for future stress physiology research and biotechnological applications
Source: Biotechnol Biofuels. 2015 Nov 25;8:188. doi: 10.1186/s13068-015-0381-1 (PMC4659200; doi:10.1186/s13068-015-0381-1)
Supplement: Supplementary file 12 — 10.1186/s13068-015-0381-1 Distribution of top plant GO slim categories for L. minor, S. polyrhiza, Z. mays, and O. sative proteomes. GO slim categories were assigned to L. minor by InterProscan5. GO slim categories of S. polyrhiza were extracted from Phytozome10 and O. sativa and Z. mays from Plaza 3.0. [file 13068_2015_381_MOESM12_ESM.pdf]

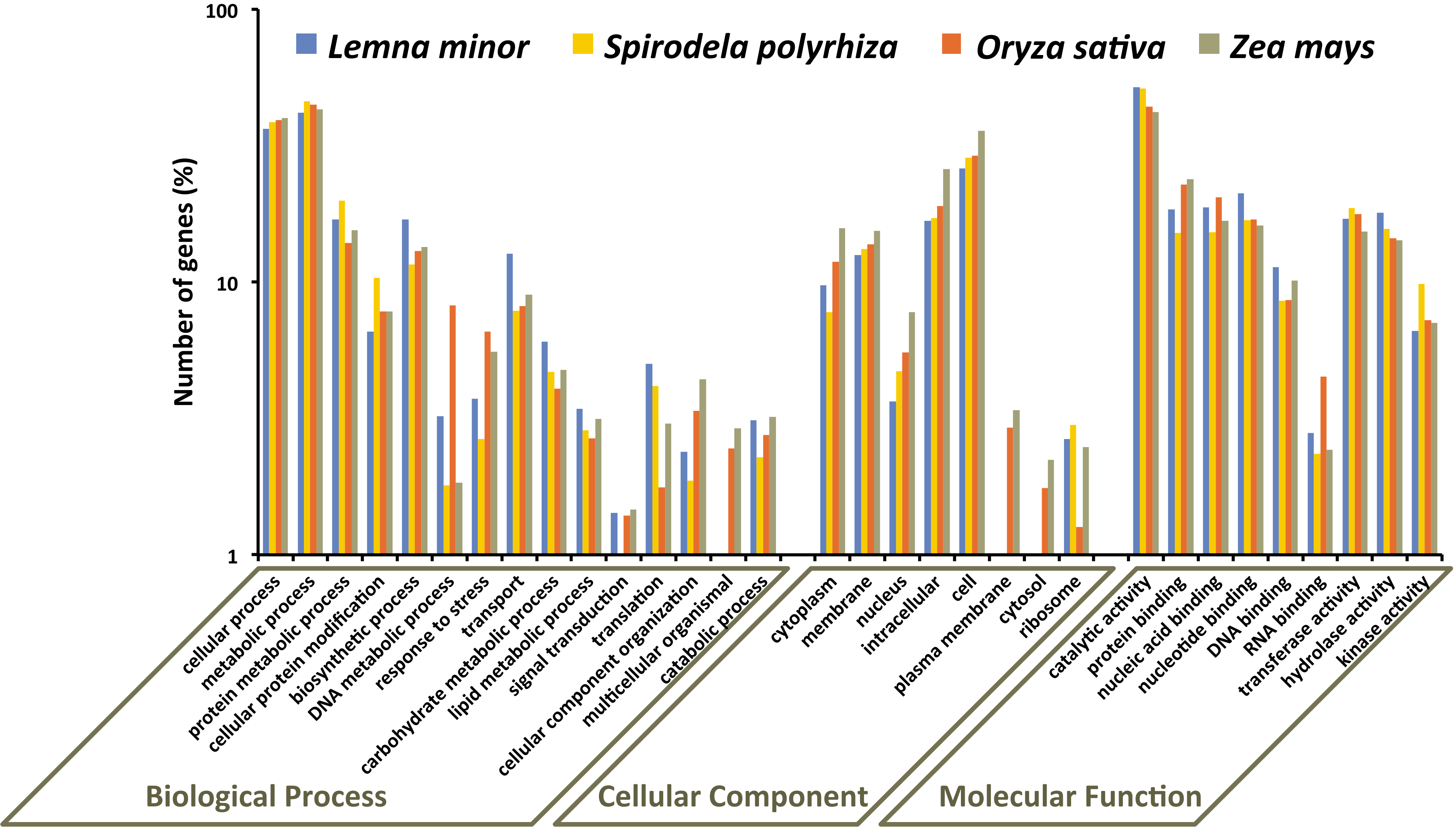

Lemna minor
Spirodela polyrhiza
Oryza sativa
Zea mays
Number of genes (%)
100
10
1
cellular process
metabolic process
protein metabolic process
cellular protein modification
biosynthetic process
DNA metabolic process
response to stress
transport
carbohydrate metabolic process
lipid metabolic process
signal transduction
translation
cellular component organization
multicellular organismal
catabolic process
cytoplasm
membrane
nucleus
intracellular
cell
plasma membrane
cytosol
ribosome
catalytic activity
protein binding
nucleic acid binding
nucleotide binding
DNA binding
RNA binding
transferase activity
hydrolase activity
kinase activity
Biological Process
Cellular Component
Molecular Function
