# Supplementary material for: The first draft genome of the aquatic model plant Lemna minor opens the route for future stress physiology research and biotechnological applications
Source: Biotechnol Biofuels. 2015 Nov 25;8:188. doi: 10.1186/s13068-015-0381-1 (PMC4659200; doi:10.1186/s13068-015-0381-1)
Supplement: Supplementary file 13 — 10.1186/s13068-015-0381-1 Overview of gene ontology classification. [file 13068_2015_381_MOESM13_ESM.docx]

**Supplementary Table S10:** overview of gene ontology classification

|  |  |  |  |  |  |  |  |  |  |  |  |  |  |  |  |  |
| --- | --- | --- | --- | --- | --- | --- | --- | --- | --- | --- | --- | --- | --- | --- | --- | --- |
| \| Biological process \| *L. minor* \| *S. polyrhiza* \| *O. sativa* \| *Z. mays* \| \| --- \| --- \| --- \| --- \| --- \| \| cellular process \| 36,38 \| 38,61 \| 39,31 \| 39,83 \| \| protein metabolic process \| 16,92 \| 19,82 \| 13,91 \| 15,52 \| \| nucleobase-containing compound metabolic process \| 11,34 \| 6,25 \| 14,05 \| 9,59 \| \| cellular protein modification process \| 6,57 \| 10,38 \| 7,80 \| 7,81 \| \| biosynthetic process \| 16,94 \| 11,58 \| 12,96 \| 13,46 \| \| DNA metabolic process \| 3,22 \| 1,80 \| 8,20 \| 1,84 \| \| response to stress \| 3,74 \| 2,65 \| 6,58 \| 5,56 \| \| transport \| 12,72 \| 7,82 \| 8,18 \| 9,01 \| \| metabolic process \| 41,75 \| 46,00 \| 44,79 \| 42,96 \| \| carbohydrate metabolic process \| 6,05 \| 4,68 \| 4,07 \| 4,78 \| \| lipid metabolic process \| 3,44 \| 2,86 \| 2,68 \| 3,14 \| \| cell communication \| 0,41 \| 0,68 \| 0,91 \| 0,98 \| \| signal transduction \| 1,42 \| 0,65 \| 1,39 \| 1,46 \| \| translation \| 5,00 \| 4,15 \| 1,77 \| 3,03 \| \| cellular component organization \| 2,39 \| 1,87 \| 3,36 \| 4,41 \| \| multicellular organismal development \| 0,44 \| 0,10 \| 2,46 \| 2,90 \| \| catabolic process \| 3,11 \| 2,28 \| 2,75 \| 3,21 \| \| photosynthesis \| 0,40 \| 0,79 \| 0,38 \| 0,67 \| \| cellular homeostasis \| 1,19 \| 0,84 \| 0,64 \| 0,84 \| \| post-embryonic development \| 0,13 \|  \| 1,08 \| 1,35 \| \| embryo development \| 0,01 \| 0,02 \| 0,35 \| 0,39 \| \| cell differentiation \| 0,01 \|  \| 0,54 \| 0,69 \| \| flower development \|  \|  \| 0,27 \| 0,33 \| \| response to endogenous stimulus \| 0,35 \| 0,17 \| 1,21 \| 1,66 \| \| response to abiotic stimulus \| 0,56 \| 0,28 \| 1,81 \| 2,39 \| \| generation of precursor metabolites and energy \| 1,90 \| 0,94 \| 0,81 \| 1,41 \| \| growth \| 0,06 \|  \| 0,33 \| 0,44 \| \| cell cycle \| 0,28 \| 0,16 \| 0,56 \| 0,61 \| \| cell growth \| 0,06 \|  \| 0,29 \| 0,37 \| \| response to biotic stimulus \| 0,08 \| 0,02 \| 0,78 \| 0,87 \| \| death \| 0,01 \| 1,06 \| 0,10 \| 0,16 \| \| cell death \| 0,01 \| 1,06 \| 0,10 \| 0,16 \| \| regulation of gene expression, epigenetic \| 0,05 \| 0,06 \| 0,18 \| 0,20 \| \| fruit ripening \|  \|  \| 0,01 \| 0,01 \| \| cell-cell signaling \|  \|  \| 0,02 \| 0,04 \| \| reproduction \| 0,43 \| 0,61 \| 1,49 \| 1,76 \| \| response to external stimulus \| 0,18 \| 0,04 \| 0,46 \| 0,47 \| \| pollination \| 0,29 \| 0,61 \| 0,52 \| 0,44 \| \| anatomical structure morphogenesis \| 0,06 \|  \| 0,69 \| 0,84 \| \| secondary metabolic process \| 0,26 \| 0,09 \| 0,47 \| 0,53 \| \| pollen-pistil interaction \| 0,29 \| 0,61 \| 0,38 \| 0,24 \| \| response to extracellular stimulus \| 0,12 \| 0,01 \| 0,19 \| 0,25 \| |  |  |  |  |  |  |  |  |  |  |  |  |  |  |  |  |
|  |  |  |  |  |  |  |  |  |  |  |  |  |  |  |  |  |
|  |  |  |  |  |  |  |  |  |  |  |  |  |  |  |  |  |
| \| Cellular component \| *L. minor* \| *S. polyrhiza* \| *O. sativa* \| *Z. mays* \| \| --- \| --- \| --- \| --- \| --- \| \| cytoplasm \| 9,75 \| 7,76 \| 11,86 \| 15,73 \| \| membrane \| 12,58 \| 13,23 \| 13,76 \| 15,39 \| \| nucleus \| 3,65 \| 4,70 \| 5,51 \| 7,74 \| \| intracellular \| 16,77 \| 17,18 \| 18,97 \| 25,96 \| \| cell \| 26,05 \| 28,52 \| 29,04 \| 35,82 \| \| plasma membrane \| 0,40 \| 0,28 \| 2,93 \| 3,38 \| \| cytosol \| 0,26 \| 0,21 \| 1,75 \| 2,22 \| \| mitochondrion \| 0,55 \| 0,64 \| 1,86 \| 2,09 \| \| extracellular region \| 0,35 \| 0,24 \| 1,80 \| 1,92 \| \| ribosome \| 2,66 \| 2,99 \| 1,26 \| 2,48 \| \| Golgi apparatus \| 0,34 \| 0,33 \| 1,01 \| 1,43 \| \| endoplasmic reticulum \| 0,46 \| 0,71 \| 0,82 \| 1,19 \| \| cell wall \| 0,37 \| 0,57 \| 0,76 \| 0,90 \| \| external encapsulating structure \| 0,42 \| 0,72 \| 0,96 \| 0,94 \| \| vacuole \| 0,11 \| 0,02 \| 0,98 \| 1,28 \| \| plastid \| 0,11 \| 0,30 \| 2,66 \| 3,78 \| \| cytoskeleton \| 0,95 \| 0,59 \| 0,77 \| 1,37 \| \| nucleolus \| 0,06 \| 0,02 \| 0,41 \| 0,58 \| \| thylakoid \| 0,46 \| 0,60 \| 0,69 \| 1,11 \| \| peroxisome \| 0,15 \| 0,13 \| 0,27 \| 0,34 \| \| endosome \| 0,01 \|  \| 0,27 \| 0,36 \| \| extracellular space \| 0,01 \|  \| 0,13 \| 0,07 \| \| nuclear envelope \| 0,12 \| 0,14 \| 0,12 \| 0,17 \| \| nucleoplasm \| 0,59 \| 0,33 \| 0,53 \| 0,72 \| \| Lysosome \| 0,01 \|  \| 0,01 \| 0,00 \| |  |  |  |  |  |  |  |  |  |  |  |  |  |  |  |  |
| \| Molecular function \| *L. minor* \| *S. polyrhiza* \| *O. sativa* \| *Z. mays* \| \| --- \| --- \| --- \| --- \| --- \| \| catalytic activity \| 51,69 \| 51,10 \| 43,96 \| 42,00 \| \| protein binding \| 18,41 \| 15,15 \| 22,79 \| 23,77 \| \| nucleic acid binding \| 18,72 \| 15,23 \| 20,41 \| 16,73 \| \| nucleotide binding \| 21,11 \| 16,86 \| 16,91 \| 16,12 \| \| DNA binding \| 11,37 \| 8,55 \| 8,60 \| 10,10 \| \| transferase activity \| 17,06 \| 18,70 \| 17,70 \| 15,30 \| \| hydrolase activity \| 17,98 \| 15,62 \| 14,44 \| 14,23 \| \| kinase activity \| 6,63 \| 9,84 \| 7,24 \| 7,07 \| \| RNA binding \| 2,79 \| 2,34 \| 4,51 \| 2,43 \| \| binding \| 64,84 \| 52,98 \| 64,11 \| 60,14 \| \| DNA binding TF activity \| 2,40 \| 3,60 \| 3,09 \| 3,95 \| \| transporter activity \| 7,39 \| 4,83 \| 4,45 \| 4,37 \| \| carbohydrate binding \| 0,63 \| 1,13 \| 1,70 \| 1,07 \| \| chromatin binding \| 0,17 \| 0,01 \| 1,09 \| 1,67 \| \| signal transducer activity \| 0,66 \| 0,83 \| 0,87 \| 0,66 \| \| lipid binding \| 0,65 \| 0,35 \| 0,92 \| 1,24 \| \| motor activity \| 0,41 \| 0,56 \| 0,30 \| 0,45 \| \| structural molecule activity \| 2,51 \| 3,33 \| 1,45 \| 2,88 \| \| receptor activity \| 0,19 \| 0,19 \| 0,29 \| 0,24 \| \| nuclease activity \| 0,90 \| 0,82 \| 0,86 \| 0,73 \| \| translation factor activity, RNA binding \| 0,79 \| 0,52 \| 0,35 \| 0,50 \| \| enzyme regulator activity \| 1,10 \| 1,55 \| 0,93 \| 1,34 \| \| oxygen binding \| 0,02 \| 0,01 \| 0,02 \| 0,01 \| \| receptor binding \| 0,02 \| 0,02 \| 0,05 \| 0,08 \| |  |  |  |  |  |  |  |  |  |  |  |  |  |  |  |  |
|  |  |  |  |  |  |  |  |  |  |  |  |  |  |  |  |  |
|  |  |  |  |  |  |  |  |  |  |  |  |  |  |  |  |  |
|  |  |  |  |  |  |  |  |  |  |  |  |  |  |  |  |  |
|  |  |  |  |  |  |  |  |  |  |  |  |  |  |  |  |  |
|  |  |  |  |  |  |  |  |  |  |  |  |  |  |  |  |  |
|  |  |  |  |  |  |  |  |  |  |  |  |  |  |  |  |  |
|  |  |  |  |  |  |  |  |  |  |  |  |  |  |  |  |  |
|  |  |  |  |  |  |  |  |  |  |  |  |  |  |  |  |  |
|  |  |  |  |  |  |  |  |  |  |  |  |  |  |  |  |  |
|  |  |  |  |  |  |  |  |  |  |  |  |  |  |  |  |  |
|  |  |  |  |  |  |  |  |  |  |  |  |  |  |  |  |  |
|  |  |  |  |  |  |  |  |  |  |  |  |  |  |  |  |  |
|  |  |  |  |  |  |  |  |  |  |  |  |  |  |  |  |  |
|  |  |  |  |  |  |  |  |  |  |  |  |  |  |  |  |  |
|  |  |  |  |  |  |  |  |  |  |  |  |  |  |  |  |  |
|  |  |  |  |  |  |  |  |  |  |  |  |  |  |  |  |  |
|  |  |  |  |  |  |  |  |  |  |  |  |  |  |  |  |  |
|  |  |  |  |  |  |  |  |  |  |  |  |  |  |  |  |  |
|  |  |  |  |  |  |  |  |  |  |  |  |  |  |  |  |  |
|  |  |  |  |  |  |  |  |  |  |  |  |  |  |  |  |  |
|  |  |  |  |  |  |  |  |  |  |  |  |  |  |  |  |  |
|  |  |  |  |  |  |  |  |  |  |  |  |  |  |  |  |  |
|  |  |  |  |  |  |  |  |  |  |  |  |  |  |  |  |  |
|  |  |  |  |  |  |  |  |  |  |  |  |  |  |  |  |  |
|  |  |  |  |  |  |  |  |  |  |  |  |  |  |  |  |  |
|  |  |  |  |  |  |  |  |  |  |  |  |  |  |  |  |  |
|  |  |  |  |  |  |  |  |  |  |  |  |  |  |  |  |  |
|  |  |  |  |  |  |  |  |  |  |  |  |  |  |  |  |  |
|  |  |  |  |  |  |  |  |  |  |  |  |  |  |  |  |  |
|  |  |  |  |  |  |  |  |  |  |  |  |  |  |  |  |  |
|  |  |  |  |  |  |  |  |  |  |  |  |  |  |  |  |  |
|  |  |  |  |  |  |  |  |  |  |  |  |  |  |  |  |  |
|  |  |  |  |  |  |  |  |  |  |  |  |  |  |  |  |  |
|  |  |  |  |  |  |  |  |  |  |  |  |  |  |  |  |  |
|  |  |  |  |  |  |  |  |  |  |  |  |  |  |  |  |  |
|  |  |  |  |  |  |  |  |  |  |  |  |  |  |  |  |  |
|  |  |  |  |  |  |  |  |  |  |  |  |  |  |  |  |  |
