# Supplementary material for: The first draft genome of the aquatic model plant Lemna minor opens the route for future stress physiology research and biotechnological applications
Source: Biotechnol Biofuels. 2015 Nov 25;8:188. doi: 10.1186/s13068-015-0381-1 (PMC4659200; doi:10.1186/s13068-015-0381-1)
Supplement: Supplementary file 14 — 10.1186/s13068-015-0381-1 Overview of the over and underrespresented GO terms for L. minor genes compared to S. polyrhiza genes and for L. minor and duckweed specific genes in L. minor genome. The number of tandem genes for each GO term are also included. [file 13068_2015_381_MOESM14_ESM.docx]

**Supplementary Table S11:** overview of the over and underrespresented GO terms for *L. minor* genes compared to *S. polyrhiza* genes and for *L. minor* and duckweed specific genes in *L. minor* genome

|  |  |  | lemna_vs_spiro | | | lemna specific | | | duckweed specific | | | tandem genes | |
| --- | --- | --- | --- | --- | --- | --- | --- | --- | --- | --- | --- | --- | --- |
| GO ID | **GO slim name** | **GO** | **Over/Under** | **FDR** | **# genes** | **Over/Under** | **FDR** | **# genes** | **Over/Under** | **FDR** | **# genes** | **Lemna** | **Spiro** |
| GO:0008219 | cell death | BP | UNDER | 2,32E-36 | 3 |  |  |  |  |  |  | 0 | 3 |
| GO:0016301 | kinase activity | MF | UNDER | 4,22E-26 | 929 |  |  |  | UNDER | 4,93E+00 | 30 | 3 | 3 |
| GO:0006464 | cellular protein modification process | BP | UNDER | 7,88E-24 | 1036 | UNDER | 1,05E-07 | 218 | UNDER | 1,09E+03 | 41 | 0 | 0 |
| GO:0000166 | nucleotide binding | MF | OVER | 2,46E-09 | 2184 | OVER | 4,55E-11 | 692 | UNDER | 3,71E-04 | 52 | 34 | 6 |
| GO:0005515 | protein binding | MF | OVER | 1,27E-08 | 2184 | UNDER | 6,92E-64 | 444 |  |  |  | 203 | 249 |
| GO:0006091 | generation of precursor metabolites and energy | BP | OVER | 1,43E-08 | 258 | OVER | 4,45E-04 | 103 | UNDER | 3,09E+04 | 0 | 0 | 0 |
| GO:0003700 | sequence-specific DNA binding TF activity | MF | UNDER | 5,62E-07 | 373 |  |  |  | OVER | 5,83E-16 | 80 | 16 | 48 |
| GO:0016787 | hydrolase activity | MF | OVER | 2,92E-06 | 2739 |  |  |  | UNDER | 1,21E+03 | 67 | 20 | 33 |
| GO:0005215 | transporter activity | MF | OVER | 5,15E-05 | 954 | OVER | 3,87E-03 | 316 | UNDER | 8,87E+03 | 16 | 19 | 35 |
| GO:0016043 | cellular component organization | BP | OVER | 5,26E-05 | 493 | UNDER | 3,89E-07 | 89 |  |  |  | 0 | 0 |
| GO:0009908 | flower development | BP | OVER | 7,64E-05 | 24 | OVER | 4,45E-04 | 16 |  |  |  | 0 | 0 |
| GO:0015979 | photosynthesis | BP | UNDER | 2,19E-04 | 61 | UNDER | 3,20E-05 | 3 |  |  |  | 2 | 1 |
| GO:0009056 | catabolic process | BP | OVER | 2,78E-04 | 422 | OVER | 4,17E-03 | 150 | UNDER | 2,18E+02 | 2 | 0 | 0 |
| GO:0003682 | chromatin binding | MF | OVER | 2,78E-04 | 26 |  |  |  |  |  |  | 1 | 0 |
| GO:0009579 | thylakoid | CC | UNDER | 3,60E-04 | 42 | UNDER | 7,12E-04 | 2 |  |  |  | 0 | 0 |
| GO:0005198 | structural molecule activity | MF | UNDER | 6,73E-04 | 389 | UNDER | 3,99E-02 | 91 | UNDER | 1,06E+04 | 9 | 0 | 2 |
| GO:0009875 | pollen-pistil interaction | BP | UNDER | 6,73E-04 | 45 |  |  |  |  |  |  | 0 | 0 |
| GO:0006950 | response to stress | BP | OVER | 2,42E-03 | 529 | OVER | 9,89E-03 | 181 | OVER | 9,31E-05 | 50 | 0 | 9 |
| GO:0030246 | carbohydrate binding | MF | UNDER | 2,43E-03 | 104 |  |  |  |  |  |  | 9 | 0 |
| GO:0009536 | plastid | CC | UNDER | 2,88E-03 | 17 | UNDER | 1,17E-02 | 0 |  |  |  | 0 | 0 |
| GO:0005840 | ribosome | CC | UNDER | 2,88E-03 | 356 | UNDER | 1,83E-02 | 80 | UNDER | 1,34E+03 | 8 | 9 | 27 |
| GO:0009628 | response to abiotic stimulus | BP | OVER | 3,28E-03 | 86 | OVER | 3,15E-02 | 35 |  |  |  | 0 | 0 |
| GO:0009991 | response to extracellular stimulus | BP | OVER | 4,79E-03 | 19 | UNDER | 3,94E-02 | 1 |  |  |  | 0 | 0 |
| GO:0030312 | external encapsulating structure | CC | UNDER | 7,42E-03 | 65 |  |  |  |  |  |  | 0 | 0 |
| GO:0007049 | cell cycle | BP | OVER | 9,59E-03 | 92 | UNDER | 6,29E-04 | 11 |  |  |  | 0 | 2 |
| GO:0008289 | lipid binding | MF | OVER | 1,35E-02 | 82 |  |  |  |  |  |  | 0 | 0 |
| GO:0030234 | enzyme regulator activity | MF | UNDER | 1,79E-02 | 160 |  |  |  | OVER | 2,44E-07 | 37 | 0 | 0 |
| GO:0009719 | response to endogenous stimulus | BP | OVER | 2,17E-02 | 55 | UNDER | 5,63E-03 | 6 |  |  |  | 0 | 0 |
| GO:0005886 | plasma membrane | CC | OVER | 2,21E-02 | 70 |  |  |  |  |  |  | 3 | 1 |
| GO:0005783 | endoplasmic reticulum | CC | UNDER | 2,23E-02 | 70 | UNDER | 1,58E-02 | 10 |  |  |  | 1 | 2 |
| GO:0019725 | cellular homeostasis | BP | OVER | 2,23E-02 | 184 |  |  |  |  |  |  | 0 | 0 |
| GO:0005634 | nucleus | CC | UNDER | 2,36E-02 | 614 |  |  |  | OVER | 1,14E+03 | 51 | 28 | 59 |
| GO:0005829 | cytosol | CC | OVER | 3,01E-02 | 41 | OVER | 1,09E-03 | 23 |  |  |  | 0 | 0 |
| GO:0005773 | vacuole | CC | OVER | 3,29E-02 | 17 |  |  |  |  |  |  | 0 | 0 |
| GO:0009653 | anatomical structure morphogenesis | BP | OVER | 3,44E-02 | 10 |  |  |  |  |  |  | 0 | 0 |
| GO:0016049 | cell growth | BP | OVER | 3,44E-02 | 10 |  |  |  |  |  |  | 2 | 0 |
| GO:0006259 | DNA metabolic process | BP | OVER | 3,46E-02 | 350 |  |  |  |  |  |  | 1 | 0 |
| GO:0008135 | translation factor activity, RNA binding | MF | OVER | 4,88E-02 | 118 | UNDER | 3,40E-02 | 22 |  |  |  | 0 | 0 |
| GO:0005975 | carbohydrate metabolic process | BP | OVER | 4,88E-02 | 776 | OVER | 2,37E-07 | 292 | UNDER | 1,01E+03 | 14 | 43 | 55 |
| GO:0006810 | transport | BP |  |  |  | UNDER | 4,18E-10 | 96 |  |  |  | 44 | 33 |
| GO:0003677 | DNA binding | MF |  |  |  | UNDER | 5,79E-06 | 284 | OVER | 8,81E+00 | 79 | 71 | 70 |
| GO:0005739 | mitochondrion | CC |  |  |  | UNDER | 7,79E-06 | 5 |  |  |  | 0 | 0 |
| GO:0005794 | Golgi apparatus | CC |  |  |  | UNDER | 4,31E-05 | 1 |  |  |  | 2 | 0 |
| GO:0016020 | membrane | CC |  |  |  | UNDER | 3,77E-04 | 475 |  |  |  | 74 | 136 |
| GO:0006629 | lipid metabolic process | BP |  |  |  | OVER | 3,92E-04 | 182 |  |  |  | 14 | 33 |
| GO:0016740 | transferase activity | MF |  |  |  | OVER | 4,45E-04 | 692 |  |  |  | 7 | 3 |
| GO:0006412 | translation | BP |  |  |  | OVER | 1,09E-03 | 187 | UNDER | 2,16E+03 | 9 | 10 | 30 |
| GO:0006139 | NB-cont. compound MP | BP |  |  |  | OVER | 1,29E-03 | 669 | OVER | 3,75E-03 | 137 | 5 | 2 |
| GO:0005654 | nucleoplasm | CC |  |  |  | UNDER | 2,90E-03 | 6 |  |  |  | 0 | 0 |
| GO:0004518 | nuclease activity | MF |  |  |  | UNDER | 3,85E-03 | 21 |  |  |  | 0 | 3 |
| GO:0005622 | intracellular | CC |  |  |  | UNDER | 1,22E-02 | 666 |  |  |  | 11 | 40 |
| GO:0005618 | cell wall | CC |  |  |  | UNDER | 2,49E-02 | 8 |  |  |  | 8 | 21 |
| GO:0005856 | cytoskeleton | CC |  |  |  | UNDER | 3,25E-02 | 24 |  |  |  | 0 | 0 |
| GO:0007165 | signal transduction | BP |  |  |  | UNDER | 3,94E-02 | 36 |  |  |  | 7 | 9 |
| GO:0003774 | motor activity | MF |  |  |  | UNDER | 4,11E-02 | 10 |  |  |  | 2 | 3 |
| GO:0040007 | growth | BP |  |  |  |  |  |  | OVER | 3,04E-27 | 32 | 0 | 0 |
| GO:0009987 | cellular process | BP |  |  |  |  |  |  | UNDER | 1,80E+04 | 254 | 0 | 0 |
