# Supplementary material for: The first draft genome of the aquatic model plant Lemna minor opens the route for future stress physiology research and biotechnological applications
Source: Biotechnol Biofuels. 2015 Nov 25;8:188. doi: 10.1186/s13068-015-0381-1 (PMC4659200; doi:10.1186/s13068-015-0381-1)
Supplement: Supplementary file 17 — 10.1186/s13068-015-0381-1 Blast hit results of L. minor glutamine synthetase and glutamate synthase genes. [file 13068_2015_381_MOESM17_ESM.docx]

**Supplementary Table S12:** BLAST (NCBI) best hit results of *L. minor* glutamine synthetase and glutamate synthase genes

| putative function: | Lemna gene | best blast hit against (blastP, E-value < 0.05) | | | KEGG BRITE ontology | | GO term |
| --- | --- | --- | --- | --- | --- | --- | --- |
|  |  | **Uniprot reference database** | **uniprot proteins, not reviewed, A. thaliana and O. japonica** | **uniprot proteins, reviewed, A. thaliana and O. japonica** |  | |  |
| glutamine synthetase | Lminor_016485 | cDNA clone:001-119-H04, full insert sequence (Oryza sativa subsp. japonica) | GLN2 Glutamine synthetase, chloroplastic (Oryza sativa subsp. japonica) | GS2 Glutamine synthetase leaf isozyme, chloroplastic (Medicago sativa) | 0 | 0 | GO:0004356 |
| glutamine synthetase | Lminor_018039 | GLN1-4 Glutamine synthetase cytosolic isozyme 1-4 (Arabidopsis thaliana) | GLN1-4 Glutamine synthetase cytosolic isozyme 1-4 (Arabidopsis thaliana) | GLN1 Glutamine synthetase cytosolic isozyme (Lotus japonicus) | 0 | 0 | GO:0004356 |
| glutamine synthetase | Lminor_009952 | Nodulin/glutamate-ammonia ligase-like protein (Arabidopsis thaliana) | GLN1-2 Glutamine synthetase cytosolic isozyme 1-2 (Arabidopsis thaliana) | Glutamine synthetase cytosolic isozyme 2 (Glycine max) | 0 | 0 | GO:0004356 |
| glutamine synthetase | Lminor_013511 | Os02g0735200 Glutamine synthetase (Oryza sativa subsp. japonica) | GLN1-1 Glutamine synthetase cytosolic isozyme 1-1 (Oryza sativa subsp. japonica) | Glutamine synthetase cytosolic isozyme (Medicago sativa) | 0 | 0 | GO:0004356 |
| glutamine synthetase | Lminor_000396 | GLN1-1 Glutamine synthetase cytosolic isozyme 1-1 (Arabidopsis thaliana) | GLN1-1 Glutamine synthetase cytosolic isozyme 1-1 (Arabidopsis thaliana) | GLN2 Glutamine synthetase, chloroplastic (Chlamydomonas reinhardtii) | 0 | 0 | GO:0004356 |
| glutamine synthetase | Lminor_000189 | GLN1-1 Glutamine synthetase cytosolic isozyme 1-1 (Arabidopsis thaliana) | GLN1-1 Glutamine synthetase cytosolic isozyme 1-1 (Arabidopsis thaliana) | GLN2 Glutamine synthetase, chloroplastic (Chlamydomonas reinhardtii) | 0 | 0 | GO:0004356 |
| glutamine synthetase | Lminor_021806 | GLN1-3 Glutamine synthetase cytosolic isozyme 1-3 (Arabidopsis thaliana) | GLN1-3 Glutamine synthetase cytosolic isozyme 1-3 (Arabidopsis thaliana) | GLN2 Glutamine synthetase, chloroplastic (Chlamydomonas reinhardtii) | 0 | 0 | GO:0004356 |
| glutamine synthetase | Lminor_002807 | GLN2 Glutamine synthetase, chloroplastic (Oryza sativa subsp. japonica) | GLN2 Glutamine synthetase, chloroplastic (Oryza sativa subsp. japonica) | Glutamine synthetase leaf isozyme, chloroplastic (Phaseolus vulgaris) | K01915 | glutamine synthetase [EC:6.3.1.2] | GO:0004356 |
| glutamine synthetase | Lminor_011731 | Os02g0735200 Glutamine synthetase (Oryza sativa subsp. japonica) | GLN1-1 Glutamine synthetase cytosolic isozyme 1-1 (Oryza sativa subsp. japonica) | Glutamine synthetase nodule isozyme (Vigna aconitifolia) | 0 | 0 | GO:0004356 |
| glutamine synthetase | Lminor_010860 | At3g53180 Nodulin / glutamate-ammonia ligase-like protein (Arabidopsis thaliana) | GLN1-2 Glutamine synthetase cytosolic isozyme 1-2 (Arabidopsis thaliana) | Glutamine synthetase cytosolic isozyme 2 (Glycine max) | K01915 | glutamine synthetase [EC:6.3.1.2] | GO:0004356 |
| glutamine synthetase | Lminor_004908 | Os02g0735200 Glutamine synthetase (Oryza sativa subsp. japonica) | GLN1-1 Glutamine synthetase cytosolic isozyme 1-1 (Oryza sativa subsp. japonica) | GS1-2 Glutamine synthetase cytosolic isozyme 2 (Vitis vinifera) | K01915 | glutamine synthetase [EC:6.3.1.2] | GO:0004356 |
| glutamine synthetase | Lminor_013594 | Os02g0735200 Glutamine synthetase (Oryza sativa subsp. japonica) | GLN1-1 Glutamine synthetase cytosolic isozyme 1-1 (Oryza sativa subsp. japonica) | Glutamine synthetase cytosolic isozyme (Medicago sativa) | 0 | 0 | GO:0004356 |
|  |  |  |  |  |  |  |  |
|  |  |  |  |  |  |  |  |
| glutamate synthase | Lminor_004998 | Os05g0555600 Glutamate synthase 2 [NADH], chloroplastic (Oryza sativa subsp. japonica) | Os05g0555600 Glutamate synthase 2 [NADH], chloroplastic (Oryza sativa subsp. japonica) | Os05g0555600 Glutamate synthase 2 [NADH], chloroplastic (Oryza sativa subsp. japonica) | K00284 | glutamate synthase (ferredoxin) [EC:1.4.7.1] | GO:0006537 |
| glutamate synthase | Lminor_007141 | Os05g0555600 Glutamate synthase 2 [NADH], chloroplastic (Oryza sativa subsp. japonica) | Os05g0555600 Glutamate synthase 2 [NADH], chloroplastic (Oryza sativa subsp. japonica) | Os05g0555600 Glutamate synthase 2 [NADH], chloroplastic (Oryza sativa subsp. japonica) | K00265 | glutamate synthase (NADPH/NADH) large chain [EC:1.4.1.13 1.4.1.14] | GO:0006537 |
| glutamate synthase | Lminor_010625 | GLT1 Glutamate synthase 1 [NADH], chloroplastic (Arabidopsis thaliana) | GLT1 Glutamate synthase 1 [NADH], chloroplastic (Arabidopsis thaliana) | Glutamate synthase [NADH], amyloplastic (Medicago sativa) | 0 | 0 | GO:0006537 |
| glutamate synthase | Lminor_009465 | OsJ_19492 Putative uncharacterized protein (Oryza sativa subsp. japonica) | Os05g0555600 Glutamate synthase 2 [NADH], chloroplastic (Oryza sativa subsp. japonica) | Os05g0555600 Glutamate synthase 2 [NADH], chloroplastic (Oryza sativa subsp. japonica) | K00266 | glutamate synthase (NADPH/NADH) small chain [EC:1.4.1.13 1.4.1.14] | GO:0006537 |
| glutamate synthase | Lminor_011206 | GLT1 Glutamate synthase 1 [NADH], chloroplastic (Arabidopsis thaliana) | GLT1 Glutamate synthase 1 [NADH], chloroplastic (Arabidopsis thaliana) | Glutamate synthase [NADH], amyloplastic (Medicago sativa) | 0 | 0 | GO:0006537 |
| glutamate synthase | Lminor_000523 | Os01g0681900 Glutamate synthase 1 [NADH], chloroplastic (Oryza sativa subsp. japonica) | Os01g0681900 Glutamate synthase 1 [NADH], chloroplastic (Oryza sativa subsp. japonica) | Glutamate synthase [NADH], amyloplastic (Medicago sativa) | K00284 | glutamate synthase (ferredoxin) [EC:1.4.7.1] | GO:0006537 |
| glutamate synthase | Lminor_010490 | GLU1 Ferredoxin-dependent glutamate synthase 1, chloroplastic/mitochondrial (Arabidopsis thaliana) | GLU1 Ferredoxin-dependent glutamate synthase 1, chloroplastic/mitochondrial (Arabidopsis thaliana) | FdGOGAT Ferredoxin-dependent glutamate synthase, chloroplastic (Spinacia oleracea) | 0 | 0 | GO:0006537 |
| glutamate synthase | Lminor_020569 | GLT1 Glutamate synthase 1 [NADH], chloroplastic (Arabidopsis thaliana) | GLT1 Glutamate synthase 1 [NADH], chloroplastic (Arabidopsis thaliana) | GLT1 Glutamate synthase 1 [NADH], chloroplastic (Arabidopsis thaliana) | K00284 | glutamate synthase (ferredoxin) [EC:1.4.7.1] | GO:0006537 |
| glutamate synthase | Lminor_019778 | GLU1 Ferredoxin-dependent glutamate synthase 1, chloroplastic/mitochondrial (Arabidopsis thaliana) | GLU1 Ferredoxin-dependent glutamate synthase 1, chloroplastic/mitochondrial (Arabidopsis thaliana) | GLU1 Ferredoxin-dependent glutamate synthase 1, chloroplastic/mitochondrial (Arabidopsis thaliana) | 0 | 0 | GO:0006537 |
| glutamate synthase | Lminor_014566 | Os01g0681900 Glutamate synthase 1 [NADH], chloroplastic (Oryza sativa subsp. japonica) | Os01g0681900 Glutamate synthase 1 [NADH], chloroplastic (Oryza sativa subsp. japonica) | Glutamate synthase [NADH], amyloplastic (Medicago sativa) | K00284 | glutamate synthase (ferredoxin) [EC:1.4.7.1] | GO:0006537 |
| glutamate synthase | Lminor_013798 | GLT1 Glutamate synthase 1 [NADH], chloroplastic (Arabidopsis thaliana) | GLT1 Glutamate synthase 1 [NADH], chloroplastic (Arabidopsis thaliana) | Glutamate synthase [NADH], amyloplastic (Medicago sativa) | K00284 | glutamate synthase (ferredoxin) [EC:1.4.7.1] | GO:0006537 |
| glutamate synthase | Lminor_004451 | GLT1 Glutamate synthase 1 [NADH], chloroplastic (Arabidopsis thaliana) | GLT1 Glutamate synthase 1 [NADH], chloroplastic (Arabidopsis thaliana) | GLT1 Glutamate synthase 1 [NADH], chloroplastic (Arabidopsis thaliana) | K00284 | glutamate synthase (ferredoxin) [EC:1.4.7.1] | GO:0006537 |
| glutamate synthase | Lminor_021252 | GLT1 Glutamate synthase 1 [NADH], chloroplastic (Arabidopsis thaliana) | GLT1 Glutamate synthase 1 [NADH], chloroplastic (Arabidopsis thaliana) | Glutamate synthase [NADH], amyloplastic (Medicago sativa) | K00284 | glutamate synthase (ferredoxin) [EC:1.4.7.1] | GO:0006537 |
| glutamate synthase | Lminor_009493 | Os01g0681900 Glutamate synthase 1 [NADH], chloroplastic (Oryza sativa subsp. japonica) | Os01g0681900 Glutamate synthase 1 [NADH], chloroplastic (Oryza sativa subsp. japonica) | Os01g0681900 Glutamate synthase 1 [NADH], chloroplastic (Oryza sativa subsp. japonica) | K00284 | glutamate synthase (ferredoxin) [EC:1.4.7.1] | GO:0006537 |
| glutamate synthase | Lminor_010476 | GLT1 Glutamate synthase 1 [NADH], chloroplastic (Arabidopsis thaliana) | GLT1 Glutamate synthase 1 [NADH], chloroplastic (Arabidopsis thaliana) | Glutamate synthase [NADH], amyloplastic (Medicago sativa) | 0 | 0 | GO:0006537 |
| glutamate synthase | Lminor_003932 | GLU1 Ferredoxin-dependent glutamate synthase 1, chloroplastic/mitochondrial (Arabidopsis thaliana) | GLU1 Ferredoxin-dependent glutamate synthase 1, chloroplastic/mitochondrial (Arabidopsis thaliana) | FdGOGAT Ferredoxin-dependent glutamate synthase, chloroplastic (Spinacia oleracea) | K00266 | glutamate synthase (NADPH/NADH) small chain [EC:1.4.1.13 1.4.1.14] | GO:0006537 |
| glutamate synthase | Lminor_015820 | Os05g0555600 Glutamate synthase 2 [NADH], chloroplastic (Oryza sativa subsp. japonica) | Os05g0555600 Glutamate synthase 2 [NADH], chloroplastic (Oryza sativa subsp. japonica) | Os05g0555600 Glutamate synthase 2 [NADH], chloroplastic (Oryza sativa subsp. japonica) | K00284 | glutamate synthase (ferredoxin) [EC:1.4.7.1] | GO:0006537 |
| glutamate synthase | Lminor_002189 | Os05g0555600 Glutamate synthase 2 [NADH], chloroplastic (Oryza sativa subsp. japonica) | Os05g0555600 Glutamate synthase 2 [NADH], chloroplastic (Oryza sativa subsp. japonica) | Os05g0555600 Glutamate synthase 2 [NADH], chloroplastic (Oryza sativa subsp. japonica) | K00284 | glutamate synthase (ferredoxin) [EC:1.4.7.1] | GO:0006537 |
| glutamate synthase | Lminor_012735 | Os05g0555600 Glutamate synthase 2 [NADH], chloroplastic (Oryza sativa subsp. japonica) | Os05g0555600 Glutamate synthase 2 [NADH], chloroplastic (Oryza sativa subsp. japonica) | Os05g0555600 Glutamate synthase 2 [NADH], chloroplastic (Oryza sativa subsp. japonica) | K00284 | glutamate synthase (ferredoxin) [EC:1.4.7.1] | GO:0006537 |
| glutamate synthase | Lminor_009464 | Os05g0555600 Glutamate synthase 2 [NADH], chloroplastic (Oryza sativa subsp. japonica) | Os05g0555600 Glutamate synthase 2 [NADH], chloroplastic (Oryza sativa subsp. japonica) | Glutamate synthase [NADH], amyloplastic (Medicago sativa) | 0 | 0 | GO:0006537 |
| glutamate synthase | Lminor_002530 | GLT1 Glutamate synthase 1 [NADH], chloroplastic (Arabidopsis thaliana) | GLT1 Glutamate synthase 1 [NADH], chloroplastic (Arabidopsis thaliana) | GLT1 Glutamate synthase 1 [NADH], chloroplastic (Arabidopsis thaliana) | K00264 | glutamate synthase (NADPH/NADH) [EC:1.4.1.13 1.4.1.14] | GO:0006537 |
